# Understanding Periodic and Non-periodic Chemistry in Periodic Tables

**DOI:** 10.3389/fchem.2020.00813

**Published:** 2021-01-06

**Authors:** Changsu Cao, René E. Vernon, W. H. Eugen Schwarz, Jun Li

**Affiliations:** ^1^Department of Chemistry, Tsinghua University, Beijing, China; ^2^Charles Sturt University, Wagga Wagga, NSW, Australia; ^3^Department of Chemistry, University of Siegen, Siegen, Germany; ^4^Department of Chemistry, Southern University of Science and Technology, Shenzhen, China

**Keywords:** chemical elements, chemical properties, electron configurations, orbital energies, orbital radii, periodic tables, relativistic effects, superheavy elements

## Abstract

The chemical elements are the “conserved principles” or “kernels” of chemistry that are retained when substances are altered. Comprehensive overviews of the chemistry of the elements and their compounds are needed in chemical science. To this end, a graphical display of the chemical properties of the elements, in the form of a Periodic Table, is the helpful tool. Such tables have been designed with the aim of either classifying real chemical substances or emphasizing formal and aesthetic concepts. Simplified, artistic, or economic tables are relevant to educational and cultural fields, while practicing chemists profit more from “chemical tables of chemical elements.” Such tables should incorporate four aspects: **(i)** typical valence *electron configurations of bonded atoms* in chemical compounds (instead of the common but chemically atypical ground states of free atoms in physical vacuum); **(ii)** at least three basic chemical properties (*valence number, size, and energy* of the valence shells), their joint variation across the elements showing principal and secondary periodicity; **(iii)** elements in which the (sp)^8^, (d)^10^, and (f)^14^ *valence shells become closed and inert under ambient chemical conditions*, thereby determining the “fix-points” of chemical periodicity; **(iv)** *peculiar elements at the top and at the bottom* of the Periodic Table. While it is essential that Periodic Tables display important trends in element chemistry we need to keep our eyes open for unexpected chemical behavior in ambient, near ambient, or unusual conditions. The combination of experimental data and theoretical insight supports a more nuanced understanding of complex periodic trends and non-periodic phenomena.

## Introduction

Two to one-and-half centuries ago, authors of chemistry books and chemistry teachers such as Leopold Gmelin (Gmelin, 1843), Lothar Meyer (Meyer, 1864), Dmitri Mendeleev (Mendeleev, 1869b) and Viktor von Richter (Von Richter, 1875) felt the need for an ordered arrangement of the increasing number of elements. They addressed this need with the help of two-dimensional tables for groups of elements. Within half a century, with more or less delay depending on the author, Periodic Tables of elements entered most chemistry books and lecture rooms (Kaji et al., 2015; Robinson, 2019).

Then, during the past hundred years, students learned general and inorganic chemistry, and later practiced these through Periodic-Table colored glasses, rationalized by atomic structure theory. Thus, modern chemistry developed not only along the lines of easily available and practically useful chemicals, but also with effectively blinkered expectations according to the Periodic Table (Keserü et al., 2014; Pye et al., 2017; Llanos et al., 2019; Restrepo, 2019a,b). Under such circumstances, misunderstandings of the Periodic Table happen easily, and unexpected chemistry is overlooked. Some compounds or chemical preparation methods were thought to be non-existent or impossible. Therefore, we analyze the following points: the general principles of empirical periodicity; their objective physical background; deviations from expected periodicity; misrepresentations or misinterpretations of periodicity; and unexpected trends in chemistry. These points are illustrated with examples. Before the (sub)sections we present the inferences according to our own viewpoints, as *take-home messages in italicized text*. The general conclusions are presented in the last, summarizing section.

## The Emergence of Naturally 2-Dimensional Tables of Chemical Elements

*Chemical elements are the basic, abstract entities conserved in chemical transformations of real substances. The many allotropic ‘elementary substances’ (carbon as diamond, graphite, graphenes, nanotubes, fullerenes, etc., for example) are composed of a single ‘abstract element’ only. The IUPAC suggests using the word ‘element’ as a homonym for both. Common Periodic Tables are mnemonics for the trends of the meta-properties of the chemical elements under common conditions, useful in practical chemistry and in chemical education. The chemical ordinal number Z of an element in the Periodic Table is equal to the physical cardinal number of Z electrons in the neutral atom around its nucleus of the same charge number Z*.

### History

Chemistry is the art, craft, and science of modifying matter, hopefully improving materials for the benefits of humanity. Most chemical materials are used *under ambient conditions*, which is the most important aspect of chemistry for us humans. Different chemical behaviors under astrochemical or geochemical conditions may be relevant in other contexts (Esteban et al., 2004; McSween and Huss, 2010; Misra, 2012; Dong et al., 2015; Yamamoto, 2017; White, 2018; Rahm et al., 2019) and may suggest differently designed Periodic Tables.

Various notions of ‘origins,’ ‘principles,’ or ‘elements’ of the material world have been developed since antiquity. By the term ‘chemical element’ we are referring to an immutable something (a conservation principle in the physical sense) that is preserved in chemical transmutations from one chemical material to another. Our present understanding has arisen since the late eighteenth century. Even now there are still open questions (Ghibaudi et al., 2013; Scerri and Ghibaudi, 2020). The concept of an element has three basic aspects. Until the advent of the Renaissance and Enlightenment in sixteenth century Europe, elements were regarded in all developed cultures as carriers of directly observable qualities. This concept has survived only with a secondary bearing. For example, atomic weight and atomic volume were instrumental in the early development of Periodic Tables by Mendeleev and Meyer. And the density of an element, given by atomic weight divided by atomic volume, influences the observable densities of the compounds of that element.

However, the rational and enlightened Greek philosophy of the period two to one-half millennia ago was unique in human conceptual development. Sage thinkers suggested, for example, two atomistic concepts of the elements. Demokritos, Epikouros, and Titus Lucretius Carus wrote of conserved particles, forming compounds that induce the observations in our senses. Lucretius discussed many examples of natural experiences from daily life, craftsmen and doctors, and similar ideas still form the basis of present chemical atomism. Platon developed the first speculative “mathematical sub-atomic theory” that was known to the inventors of quantum mechanics and in this sense survives in modern subatomic physics (Heisenberg, 1959; Von Weizsäcker, 1971, 1985; Stückelberger, 1979; Grimes, 1983; Metzger, 1983).

Anyway, the concept of *one abstract conserved chemical element* should always and explicitly be distinguished from the *many* allotropes and phases of *real transformable elementary substances*, consisting of a single abstract element only (Van Spronsen, 1969; Scerri, 2007, 2020; Cao et al., 2019). For example, we distinguish between carbon as the abstract element found in carbon dioxide (CO_2_) and such allotropic forms of phases of pure carbon as diamond, graphite, the many different graphenes, nanotubes and fullerenes, and amorphous soot. At present more than a hundred (i.e., 118) elements, are known, without a gap.

The modern concept of conserved elements in chemical reactions was put into reality in the ‘chemical revolution’ by a network of scientists in Paris around the couple of Antoine-Laurent de Lavoisier and Marie-Anne Pierrette Paulze, a decade ahead of the cultural and political revolution in France (Ihde, 1964; Brock, 1992; Scerri, 2007, 2020). When the first half-hundred elements had been discovered around 1820, the need for *a systematic ordering and a classification* (Leal and Restrepo, 2019) became pressing. An early two-dimensional arrangement of elements was propagated in Leopold Gmelin's Handbooks (e.g., Gmelin, 1843), based purely on qualitative chemical experiences.

At the very first international scientific congress at Karlsruhe in 1860, Cannizzaro promoted older physico-chemical concepts, which permitted the change-over *from qualitative to quantitative criteria*. First, the elements could be linearly ordered according to the unique semi-empirical atomic weight numbers, instead of the partial ordering with the help of purely empirical equivalent weights, or of compounds' densities. Second, the elements could be classified into similarity groups, on the basis of oxidation and valence numbers (Meyer, 1864) or unique sum formulas (Mendeleev, 1869a), and atomic volume values (Meyer, 1870), in addition to general qualitative chemical experience (Scerri, 2007, 2020; Gade, 2019).

On the basis of the Geiger-Marsden-Rutherford experiments of atomic scattering of α- and β-particles in the years of 1908 to 1913, Rutherford concluded (first in 1911) that atoms consist of a tiny massive center of *positive charge of ca. half its mass number*, and a surrounding cloud of a respective number of negative electrons. Van den Broek, a scientifically interested amateur immediately suggested the nuclear charge and electron number should be a bit smaller and equal to the element number in the Periodic Table. Thereby he attached a well-defined physical meaning to this so-far, somewhat arbitrary, chemical number. This development inspired Moseley in his experimental X-ray spectroscopic work on the elements. He could prove that the chemically motivated order of elements cobalt < nickel has a physical basis, while the average atomic weights *(A)* of the elements from the earth's crust (which have a somewhat accidental origin in cosmic and geochemical history) increase in the opposite order, nickel *(A* = 58.7) to cobalt *(A* = 58.9). Later, also the order of argon *(A* = 39.95) < potassium *(A* = 39.1) and of tellurium *(A* = 127.6) < iodine *(A* = 126.9) was verified (Da Costa Andrade, 1958; Scerri, 2007, 2020).

While Moseley's work was at first purely empirical, Bohr's invention of his atomic model in 1913 allowed Moseley to verify van den Broek's hypothesis. The elements became ordered according to the *physically based chemical element number Z*, which is the nuclear charge and electron number of the elemental atoms. Both enter the equations of time-independent quantum-mechanics, thereby physically determining ‘static’ chemistry, say at the lowest order Born-Oppenheimer approximation. Further, *Z* correlates approximately with the atomic weight *A* as ensued during cosmic history. *A* enters the time-dependent equations, thereby determining ‘kinetic’ chemistry and rotational and vibrational spectroscopies. The change from the “chemically corrected empirical *A*” to the “basic, theoretical *Z*” was a conceptual change in two senses. (i) The fuzzy combination of atomic weight, maximum oxidation state and chemical similarities to linearly order the elements in the most probable manner was replaced by the unique, integer, direct experimentally-based element number *Z* with a physical (nuclear charge number) and a chemical meaning (electron number). (ii) The question for missing entries, which had caused so much uncertainness, could be answered once for all.

### Structure of Periodic Tables

*The chemical periodicity of the elements is triggered by the closure of atomic valence shells with a supervening orbital energy gap that is comparatively large compared to primary bonding energies and thermodynamic conditions in ambient conditions of pressure p and temperature T. A significant fraction of the variation of chemical behavior of the abstract elements at common conditions can be simulated by just two ‘main factors,’ consistent with the two-dimensional topology of common rectangular Periodic Tables, with the noble gases at the borders. A large part of the periodic structure will fade away at higher than common p and T, while at low T and p the diversity of chemistry increases as various new molecules can survive and, while there are fewer thermally induced reactions, a novel chemistry can be enabled by designed binding. The Periodic Rule is specific for the selected field of chemistry such as at ambient human (or planetary core, or cosmic space) conditions*.

**Figure 1:**
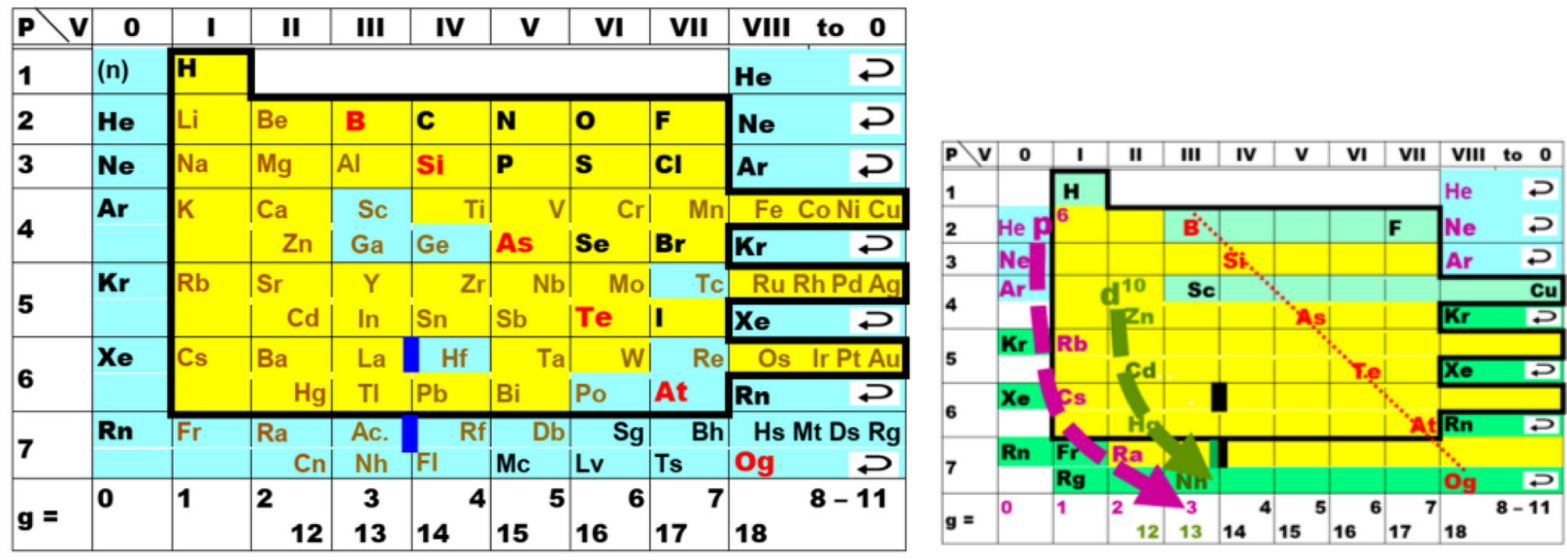
Periodic Table of elements in the ‘short’ form, as rather common during the early decades. The noble gases are here displayed twice, at the left and right borders, to underline the spiral topology of the natural system. P = number of the period, V = valence number of the group, g = modern group number. Elements known in the early 1860s are displayed within the bold frame on a yellow foreground. **(Left)** The then unknown elements are on an aqua foreground. The lanthanoids and actinoids are indicated by bold deep-blue bars (only six of the 30 were known: La, Ce, Er, Tb; Th, U). The approximate divide (bold red letters) between the more metallic (in brown) and the more non-metallic elements (in black). **(Right)** The small table highlights two points of chemical relevance. (i) Both, the well-known elements with a light green foreground toward the top of the table (H; B-F; Sc-Cu), and the more or less well-known elements with a darker green foreground toward the bottom (Kr, Xe, Rn, Og; Fr; Rg-Og) show rather ‘unique’ properties. (ii) The pivots of periodicity are the closures of the p^6^ shells of the elements in lilac (He, Ne, Ar, Rb, Cs, Ra) and of the d^10^ shells of the elements in olive (Zn, Cd, Hg, Nh). The shell closure shifts to the right toward the bottom of the table, for p^6^ from group 0 to 3, and for d^10^ from group 12 to 14, as indicated by the bold bent dashed arrows.

One of the various types of Periodic Table designs is the ‘short form,’ an example being displayed in Figure 1. In 1870, the elements with a yellow foreground were known. The *chemical periodicity* is mainly connected to the large change of chemical character from the halogens (fluorine to iodine) in group VII (or 17) through the noble gases in group VIII (or 18/0) to the alkali metals (lithium to cesium) in group I (or 1). As we now know, the halogens have a compact, strongly electron-attracting (electronegative) open valence p-shell.^1^ This becomes a closed core shell, fully occupied and chemically inert, for the alkali metals, which in addition have a new, diffuse, weakly electron-binding (electropositive) open valence s-shell, with *a large energy gap* between the (*n*−1)p and *n*s shells (Longuet-Higgins, 1957; Wang and Schwarz, 2009).

The noble gases were discovered in the 1890s (Ar was isolated in 1894, He in 1895, Ne, Kr, Xe in 1898). In principle they could be easily incorporated into the Periodic Table. Meyer's table of 1864 had columns for valences 4, 3, 2, 1 of the electronegative elements (for the C, N, O, F groups), and for valences 1, 2 of the electropositive elements (for the alkali and alkaline earth metals), but with no in-between zero valence. (Known elements B, Al, Y, La with valence 3 appeared too diverse to place them together into the equivalent of present groups 3 and 13.) Not everyone was convinced that the concept of zero-valence elements forming only elementary substances would make any sense. On the other hand, Mendeleev's tables since 1869 had eight transition groups, but only seven main groups (Figure 1), despite the gaps in the series of atomic weights between the halogens and alkali metals being large enough to insert (or not) a new group of elements. Yet, it took some time, and Mendeleev for instance did not accept the incorporation of the noble gases before 1900 (Scerri, 2007, 2020).

A somewhat less pronounced periodic jump occurs when the (*n*−1)d valence shell of the transition elements (in the nine transition groups from 3 to 11, with many colorful, multivalent, magnetic compounds from group 4 onward; see e.g., Grochala, 2020) becomes a filled and chemically inert (*n*−1)d^10^ core shell from the zinc group-12 onward.

During the times when only the yellow foreground elements in Figure 1 were known, and during the following decades when noble-gas chemistry was unknown, and when only a little chemistry of a few elements in period 7 was known, a strong conviction emerged in the chemical community, which has survived to the present day, namely: *The periodic trends in the upper part of the periodic system are valid in general*. However, we now know that the lighter elements with small principal quantum numbers of their valence shells have well-separated orbital energy bands and well-separated electron density shells in space (Jørgensen, 1969; Levine, 1970; Kohout and Savin, 1996), and can be well and easily approximated by the non-relativistic approximation of quantum theory, with negligible spin-orbit coupling. This no longer holds for the heavier atoms. Therefore, it is unrealistic to extrapolate Periodic Tables linearly and vertically down into the region of high *Z* (up to several hundred or even > 1,000; see e.g., Karol, 2002; Rath, 2018). In particular, the lynchpins of periodicity cannot be presumed to move vertically down the common designs of Periodic Tables as indicated in Figure 1, right.

**Figure 2:**
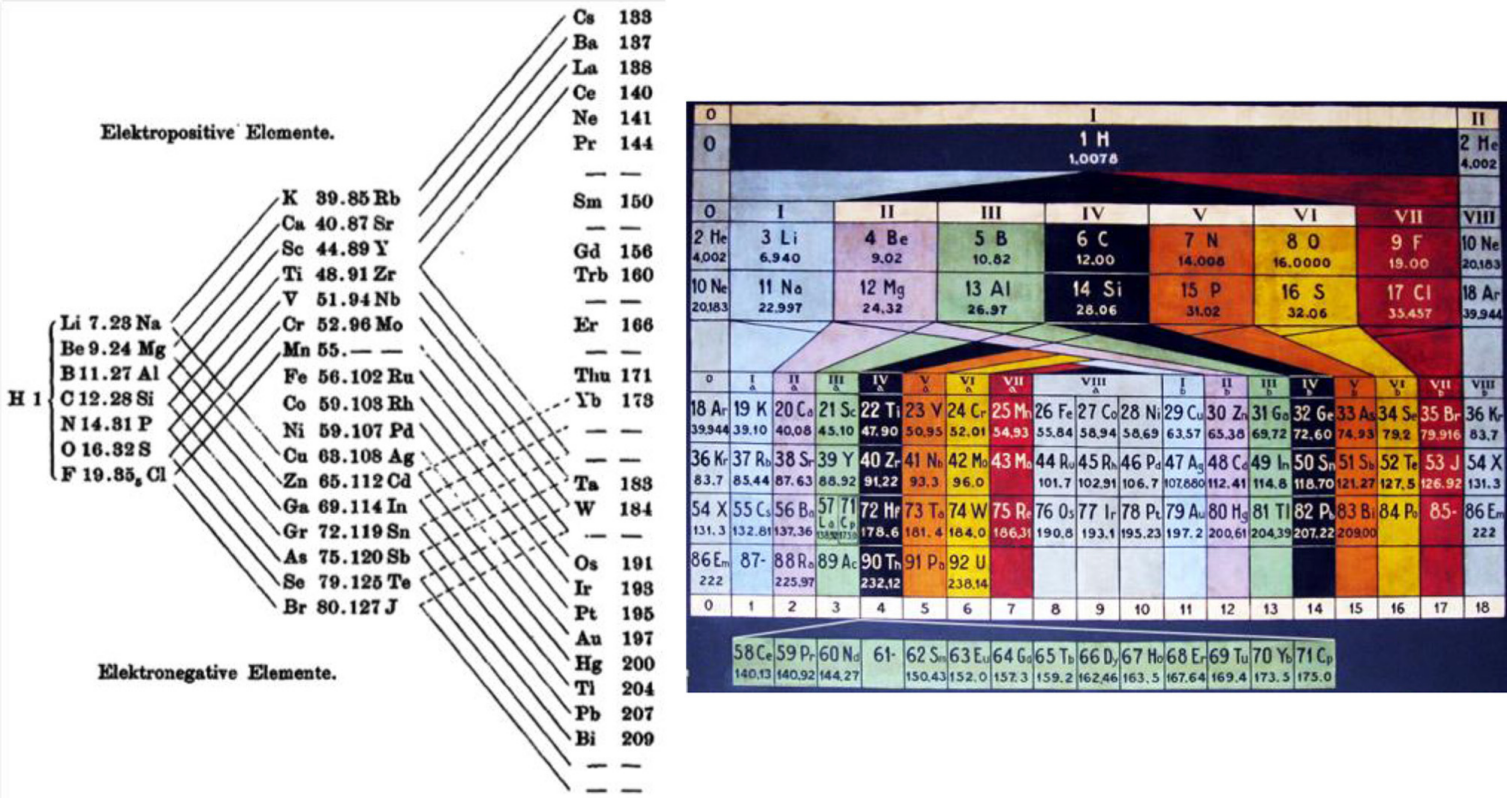
Early bifurcating Periodic Tables explicitly showing (i) the smooth trends of properties along the array of elements (from the alkali metals through the intermediate elements to the halogens), (ii) the jumps (from the halogens to the next alkali metals), and (iii) the bifurcating similarities of elements of the main and transition groups. H is positioned at the central starting point, chemical sensibly without group assignment. **(Left)** An early, bifurcating triangular table, yet without the noble gases (source: Thomsen, 1895, p. 192). **(Right)** Design of Von Antropoff (Von Antropoff, 1926), as presented in the renovated lecture hall of the University of Barcelona (source: private photo by Claudi Mans: Mans i Teixidó, 2008).

**Figure 3:**
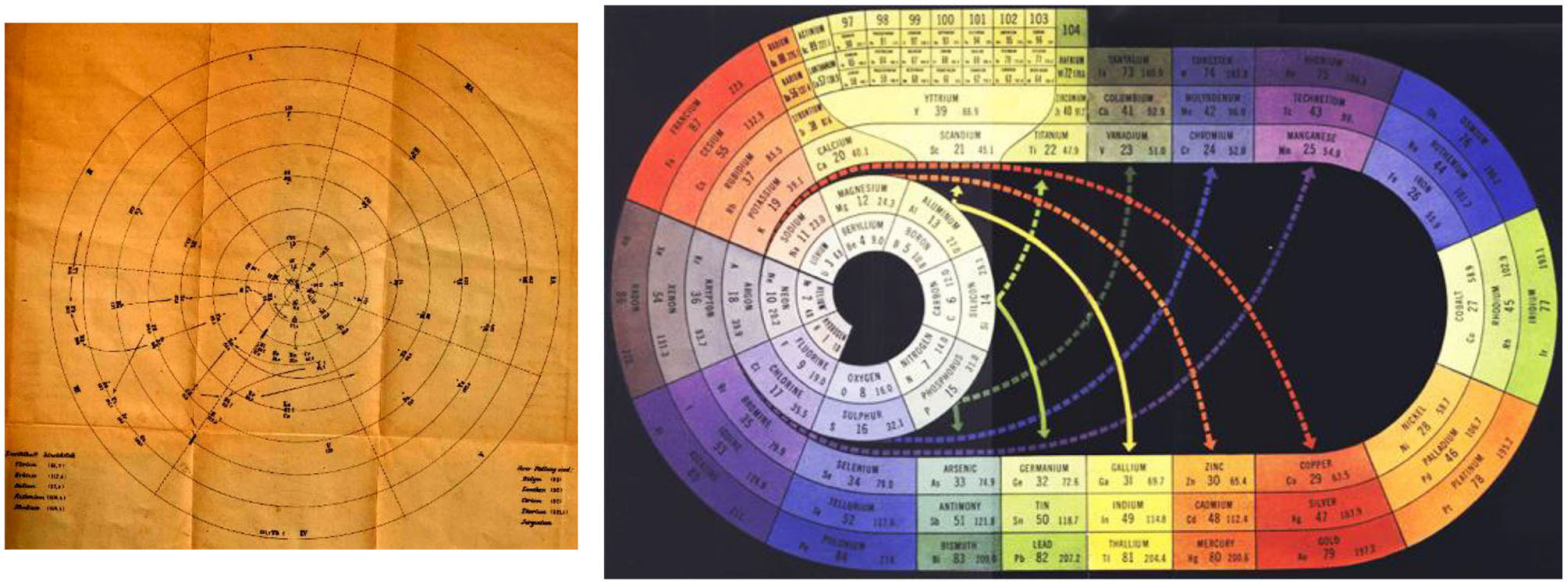
Early spiral tables stressing both the continuity of the array of elements and the bifurcation of the chemical similarity classes, as an alternative to Figure 2. **(Left)** Following Béguyer de Chancourtois (1862/3), the spiral of Baumhauer (source: Baumhauer, 1870, leaflet at the end). **(Right)** The spiral (with bifurcations) of Clark (1933, 1949, 1950) with the noble gases at the middle left (in gray) and the f-block in the boron-aluminum–scandium-yttrium group at the top left (in yellow, each f-series after La and Ac is split up into two septets: 4f in Ce^(IV,III)^-Gd^III^, Tb^(IV,III)^-Lu^III^ and 5f in Th^IV^-Cm^(IV,III)^, Bk^(IV,III)^-Lr^III^), source: Clark (1949).

**Figure 4:**
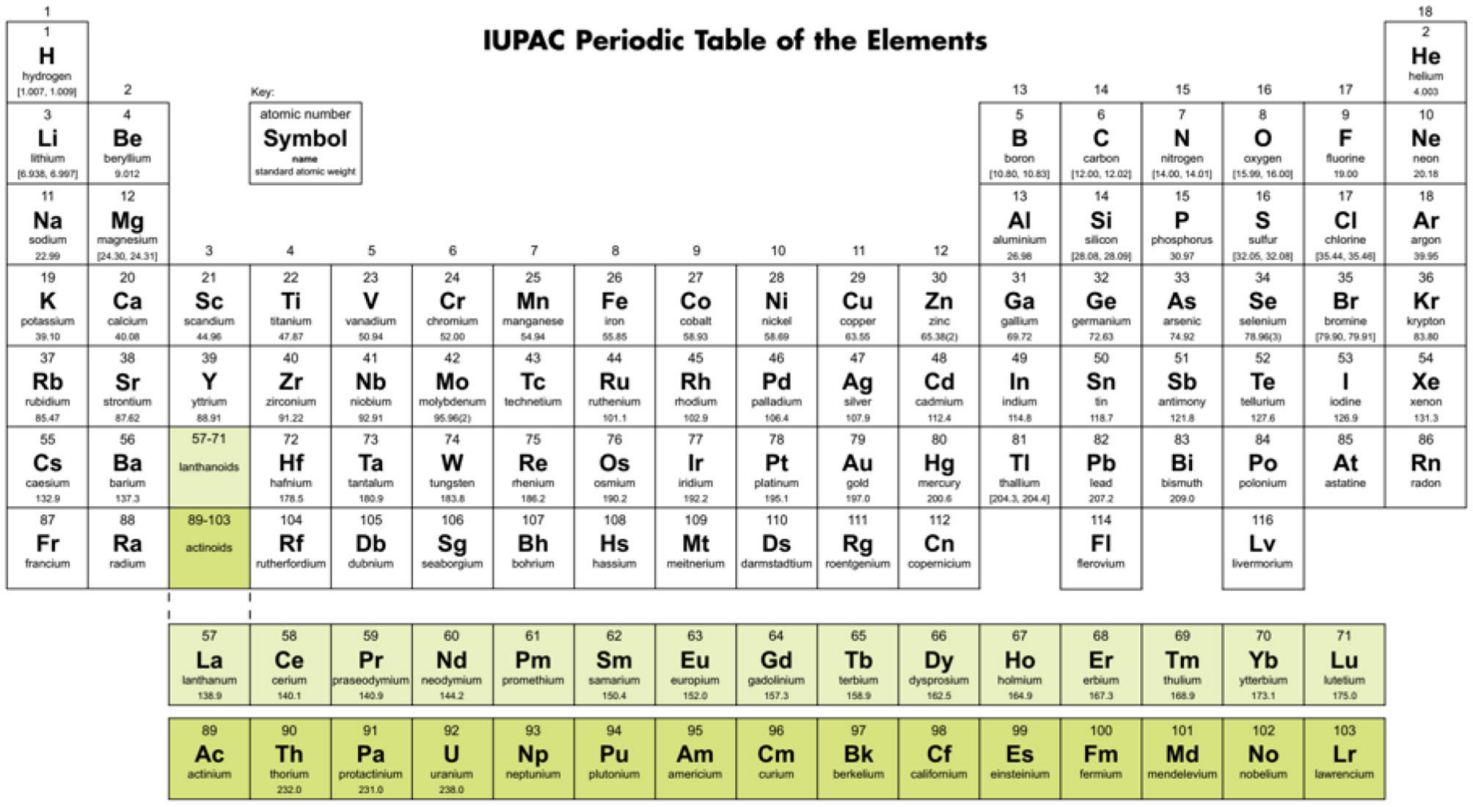
A Periodic Table in ‘medium long’ form, as suggested by the IUPAC until the end of 2015; see also the IUPAC Recommendations 2005 (Connelly et al., 2005: the Red Book III). H is positioned in group 1, He in group 18, and the f-block of 15 members still positioned so that the early members appear below their chemical relatives from the d-block (Sc, Y → La, Ac; Ti-Hf → Ce, Th; Cr-W → U; etc.). Source: IUPAC (2015).

Three important aspects were well-highlighted in some of the earlier tables (Figures 2, 3) but have unfortunately become less fashionable. **(i)** Periodicity means the repeated recurrence of properties along a coherent array; this can be underlined by repeating the border-line elements on the right and left borders. **(ii)** The first element H-1s^1^ cannot be categorically assigned to any group, neither to group 1 with 1 valence electron, nor to group 17 with 1 hole in the valence shell, nor to group 14 with half-filled valence shell, nor to more exotic suggestions such as group 3.^2^ Therefore, H is sometimes positioned on top of the whole table. **(iii)** Conversely, the light group-2 elements Be, Mg can be related to both the heavy group-2 (Ca etc.) or group-12 (Zn etc.) elements; and the light group-3 elements B and Al can be related to both the medium-heavy group-3 (Sc, Y) or group-13 (Ga etc.) elements, and Y can be related to the heavy group-3 (La, Ac) or group-3' (Lu, Lr) elements (see also Figure 4). While active chemists usually investigate the comprehensive group, there are authors who prefer to classify the border-case elements in a rigorous unique manner (Luchinskii and Trifonov, 1981; Jensen, 1982, 2003, 2015; Grochala, 2018; Petruševski and Cvetković, 2018; Chandrasekara et al., 2019; Kurushkin, 2020; Rayner-Canham, 2020; Scerri, 2020; Vernon, 2020b). Anyhow, the bifurcations in the Periodic System are a basic aspect of empirical chemical periodicity (Bayley, 1882; Carnelley, 1886; Thomsen, 1895; Bohr, 1922; Hackh, 1924; Von Antropoff, 1926; Clark, 1933, 1950; Shchukarev, 1954).

*The topology of printed pages* suggests a two-dimensional *display of the system of elements* in ‘paper format’, as initiated by Guyton de Morveau (Guyton de Morveau, 1782); Dumas (Dumas, 1828); Döbereiner (Döbereiner, 1829); and Gmelin (Gmelin, 1843). Since chemistry is much richer than any flatland projection, already Gmelin hoped that a three-dimensional matrix of elements would allow for a deeper insight into the structure of the chemistry of the elements. In the first documented periodic arrangement of all known elements, Béguyer de Chancourtois (Béguyer de Chancourtois, 1862/3) drew the one-dimensional array of elements, ordered by atomic weights, as a helix on the two-dimensional surface of a physical cylinder, embedded in our three-dimensional space. This display exhibited some of the similarities of the elements, and the smooth variation as well as jumps in their properties under ambient chemical conditions, both along the array and ‘orthogonal’ to it. Later inventors of Periodic Tables cut, so to speak, this cylindrical bent surface at different points, in order to obtain flat printable tables. A cut between the halogens and the alkali-metals, where the largest change of chemical behavior occurs, became the most favored one, at least among practicing chemists. When the noble gases were discovered, they were placed between the halogens and the alkali metals, either to the right of the halogens, or the left of the alkali metals, or both. According to each author's preferences, different rectangular (Figures 1, 2, 4, **6**) or spiral (Figure 3) or more complex graphics, flat or bent or conical or multi-connected, two-parametric tables were designed (Quam and Battell Quam, 1934; Van Spronsen, 1969; Mazurs, 1974; Scerri, 2007, 2020; Stewart, 2007, 2010; Imyanitov, 2016).

Arrangements of the elements were also suggested that are genuinely three-dimensional, that is three-parametric, not simply two-parametric ones embedded in a three-dimensional space. Conceptually there are differences between bent, variously connected two-dimensional objects such as cylindrical or spherical surfaces (e.g., cyclopolyacene, Möbius-cyclopolyacene, fullerene), three-dimensional crystal lattices and structures such as those of the zeolites. Displayed on flat paper, three-dimensional arrangements appear rather complicated, and have so far hardly impressed the chemical community. There may be good reason. Namely, comprehensive analyses of a huge number of properties of the elements and their compounds revealed just two dominant “Main Factors” and a rather large number of minor factors (Sneath, 2000; Restrepo et al., 2006; Leach, 2013).^3^ The two Main Factors, which are mixtures of electronegativity, valency, molar density, metallicity, acidity etc. simulate a significant part of the variation of the properties of the compounds of each element. Earlier work by Godovikov and Hariya (1987) continuing on Shchukarev's surveys (Shchukarev, 1969, 1977) had already produced a remarkable mapping of the broad contours of the periodic system of elements' properties, using just two criteria, an electric (ionization potential) and a spatial one (orbital radii, with a strong correlation, in periodic mapping terms, to electron affinity).

Thus, a *2-dimensional display of the elements appears naturally appropriate*, bearing in mind such periodicity is largely confined to the common conditions in our laboratories, industries and daily life. The general applicability of a simple and general *Periodic Rule* will progressively fade away, if applied to matter under more extreme conditions (as viewed from the anthropocentric standpoint). The so-called Periodic Law is a contingent rule that happens to hold in chemistry under ‘human’ conditions. Its importance in chemistry is comparable to the basic laws in physics, but its epistemological status is not comparable (Hettema and Kuipers, 1988).

### Length of Periodic Tables

*An important condition for the emergence of periodicity of chemical behavior under ambient conditions is a well-structured atomic orbital level scheme, in particular with gaps, above 1s and 2p to 6p. This quantum-mechanical phenomenon determines the period lengths of 2, 8, 8, 18, 18, 32. At the bottom of common Periodic Tables, four changes happen together, accidentally: (i) The high number Z of electrons occupy orbitals with high principal quantum numbers n, with small energy gaps. (ii) The value of the Coulomb coupling constant causes different screenings of the s, p, d, and f orbitals by the large atomic cores that smooth out the shell structure at large Z. (iii) The actual value of the fine structure constant causes additional orbital splitting of qualitative chemical relevance via spin-orbit coupling at the bottom of the table. (iv) The actual values of the coupling constants of particle physics let the nuclear lifetimes decrease at the end of the second 32-period to values below the time limit required for the existence of a chemical substance*.

Where does the *chemical system of elements end?* Physicists consider atomic nuclei as representatives of elements, and they require that the particle clusters forming a nuclear complex stay together longer than the fly-by time of ca. 10^−23^ s. On the other hand, the existence of bulk chemical stuff requires longevity of the nuclei. Lifetimes τ below a year (ca. 10^7.5^ s) will quickly cause crystal structure defects and thermo-dynamic modification. Beneficial uses approach their end, and only a few quick experiments of molecular gas-phase or surface or tracer chemistry are possible for the elements (longest isotopic lifetimes τ in parentheses): radon _86_Rn (τ ≈ 4 days), astatine _85_At (τ ≈ 1/3 day), and francium _87_Fr (τ ≈ 1/3 min); and for the late actinoids and the early transactinoids (super-heavy transition elements) up to dubnium _105_Db (Eka-Ta) with lifetimes of hours.^4^ But for the late super-heavy transition elements seaborgium _106_Sg (Eka-W) to copernicium _112_Cn (Eka-Hg) with lifetimes of minutes to seconds, and for the super-heavy p-block elements nihonium _113_Nh (Eka-Tl, τ ≈ 10 s) to oganesson _118_Og (Eka-Rn, τ < 1 ms), ultra-fast reaction-kinetics and spectroscopy come to their limits. The joint IUPAC/IUPAP definition of a chemical element is a lifetime of τ ≥ 10^−14^ s, which may be long enough for most nuclei to reach their own ground state and also to collect their atomic electrons (Wapstra, 1991). This sounds fine for nuclear and atomic physicists, though not for molecular and solid state physicists, not to speak of chemists. Accidentally in period 7, both the lifetime of the elements becomes too short from the chemical point of view, and the chemical periodicity of the electronic valence shells changes, so that period 7 represents the bottom end of the chemical periodic system (Ball, 2019).

## Emphasis of Standard Periodicity: Simplified Models for Abundant Chemistry

*The first groups of elements were recognized on qualitative chemical grounds, after the first few dozen of elements had been discovered. Quantitative values of valence, redox potential and atomic volumes (*~*radii*^3^*) established the scientific soundness of the empirically emerged Periodic Rule for chemistry under ambient conditions. The finally successful physical rationalization of the Periodic Rule was initiated by Bohr's (semi-)classical atomic model, just a century ago. The main periodic repetition of chemical properties occurs for the series of elements with n(s,p) valence shells. For periods 4 to 7, series of elements with (n*−*1)d or (n*−*2)f, (n*−*1)d valence shells appear embedded in the n(s,p) series. This yields the factual ns*|(*n*−*2)f*|*(n−1)d*|*np structure of the actual chemical periods, as an accidental by-product caused by the simple-structured physical theory, when applied to the complex field of chemistry. The series of blocks in the Periodic Table does not indicate a general order of atomic orbital energetic levels, which varies with Z and ionic charge of the atoms*.

### The Periodic Rule

The acceptance of the “*Periodic Rule*” (Figures 1–4) by the chemical community was not automatic (Gordin, 2004, 2019; Scerri, 2007, 2020). For some chemists of the time, the property-variations appeared fortuitous or partly unimpressive. However, Meyer's graphic display of periodicity of numerical atomic volumes (Meyer, 1870), and Mendeleev's correct predictions of various properties of unknown elements and their compounds by interpolation in the table (Mendeleev, 1869a; Mendelejeff, 1871: predictions on scandium, gallium, germanium—in the center of Figure 1—experimentally verified between 1875 and 1886) appeared convincing to the community (Scerri, 2007, 2020; Stewart, 2019). A theoretical breakthrough was achieved by Bohr and Coster (1923) with their (semi-)classical atomic model that reproduced the spectroscopic data of hydrogen and cationic helium (He^+^) exactly, and paved the way for a qualitative *physical rationalization of various chemical trends* (Schwarz, 2013).

From then on, in principle, the energies *and* radii (proportional to $\sqrt[{3}]{\text{volumes}}$; see Biltz, 1934) of the atomic valence *and* outer-core shells could be utilized to explain chemistry. Atomic energy levels were available from decades of atomic spectroscopy (Moore, 1949 et seq.) and atomic distances from the emerging field of X-ray crystallography (Lima-de-Faria, 1990). The new quantum mechanics of Schrödinger and Dirac was applied to *chemically unbound atoms* and reviewed by Condon and Shortley (1935). Since then it was easy to acquire basic knowledge of (i) the mixing of single-electronic *n*ℓ*j* spinor-orbital^5^ configurations in many-electronic systems, (ii) the spin-orbit mixed orbitals in the dominant (leading) configuration, and (iii) the spin-orbit-coupling that may be neglected in non-relativistic approximate atomic *n*ℓ position-orbitals (good for the lighter elements). The plethora of atomic spectral data was liberated from the data graveyard and chemically usefully interpreted (Herzberg, 1937).

### s,p vs. d,f Shells

*The energies (and radii) of the outer s and p valence shells of the elemental atoms form pairs, smoothly varying along the series of elements. This gives rise to the sp block of main-group elements with smoothly increasing electronegativity and decreasing atomic radii. The chemical periodicity is fixed by the large jump of elemental properties under ambient chemical conditions, when the 1s or nsp (n* = *2, 3, 4, 5, 6) shell becomes filled and inert, with a new loosely bound valence shell above a large energy gap at the beginning of the new period. Due to better shielding from nuclear attraction by the sp core electrons, the (n*−*1)d and (n*−*2)f shells however vary in steps along the series of elements. They fall below the sp valence band after group 2 or 3 and give rise to the transition block of d and df elements of groups 3 to 11, embedded in the sp block. In the heavier periods, divalent main group elements appear twice, with an empty d*^0^ *Rydberg*^6^ *shell in group 2 and a filled d*^10^ *core shell in group 12*.

The *smooth and parallel variation of the atomic one-electron s- and p-levels vs. the stepwise variation of the d- and f-levels, and the changing order of s and p vs. d and f* was known (in principle) since Bohr and Coster (Bohr and Coster, 1923). Bohr's (semi-)classical model concepts worked approximately even for the interpretation of the observed many-electronic atomic levels. A quarter century later, after WW2, atomic structure quantum calculations became routine. *All orbital energy levels and orbital radii* for all free neutral atoms were published by various groups. We mention a few of these groups here: Latter (Latter, 1955), Herman and Skillman (Herman and Skillman, 1963), Gombás (Gombás and Szondy, 1970), Fricke and Waber (Fricke and Waber, 1971), Desclaux (Desclaux, 1973). They found their way into few textbooks on physical chemistry, such as Glasstone (Glasstone, 1946 seq.), quantum chemistry, such as Levine (Levine, 1970), or the Periodic Table, such as Mazurs (Mazurs, 1974). (It is recommended to check the original papers cited by Mazurs as his reproductions often were ‘artistically’ redrawn).

**Figure 5:**
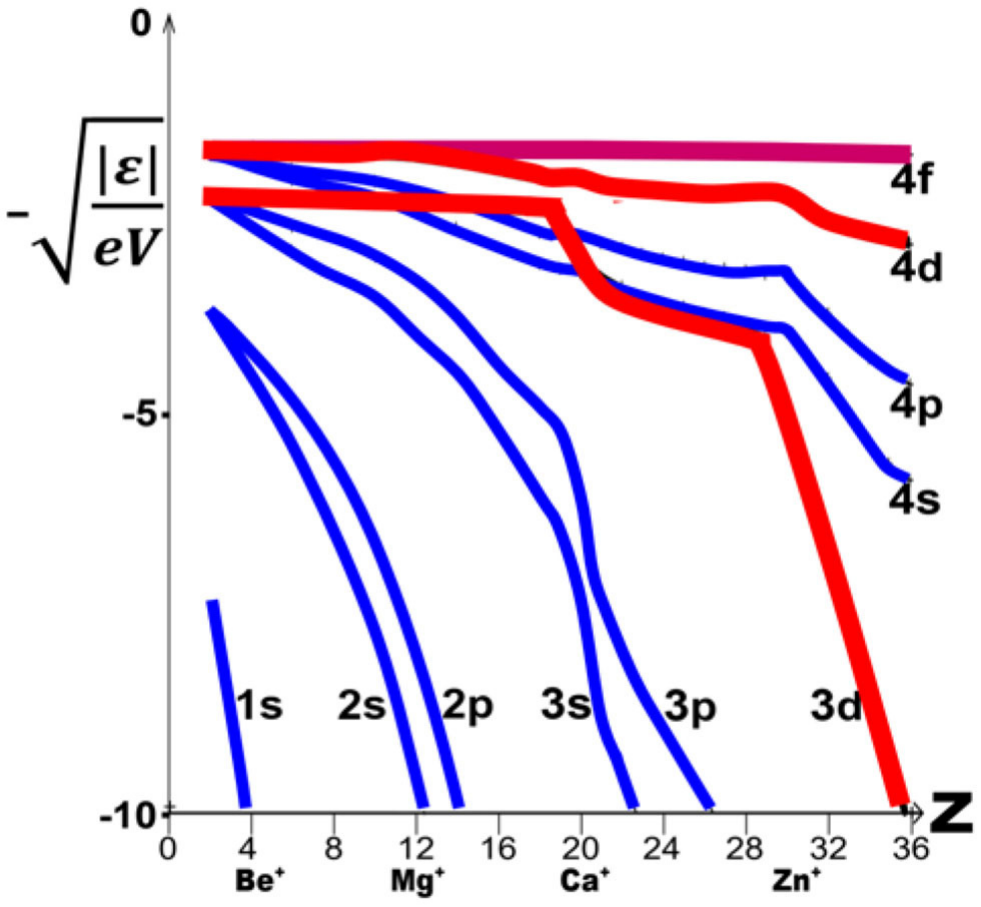
Atomic one-electron energies **|ε|** for 1s to 4f orbitals, vs. element number ***Z***. **|ε|** is defined as the experimental atomic second ionization energy (correlating with the metals' chemistries), configuration-averaged^7^ and displayed as √**|ε**/eV**|**, which corresponds to *Z*_eff_/*n*_eff_ (data from NIST: NIST Team, 2019). Note the smooth and nearly parallel decline of the *n*s and *n*p energies (in blue) vs. the more stepwise decline of the *n*d energies (in red) for increasing *Z* (and at higher **Z** also of the *n*f energies, not shown here).

The *n*s and *n*p orbitals have rather similar energies. For increasing element number *Z*, they stabilize smoothly together, with secondary kinks occurring when a new inner subshell becomes occupied (Figure 5). Since the d and f orbitals are well-shielded from nuclear attraction by the s and p core electrons, their energy levels at first hardly vary with increasing Z. For the noble gases and alkali metals in groups 0 and 1 of period *n*, there is a large energy gap between the outer closed (*n*−1)p^6^ core shell and the next *n*s, *n*p valence shells. The (*n*−1)d levels [and for the heaviest elements also the (*n*−2)f levels] are even higher in energy, but then ‘collapse’ from far above to just below the *n*s, *n*p pair (Goeppert Mayer, 1941; Connerade, 1991; Schwarz, 2010b; Cao et al., 2019). The varying order of canonical one-electron energy levels ε ^7^ along the periods of elements (Figure 5) is described by the following relations (1) to (4), where the symbol ≪ indicates atomic orbital energy differences so large that the lower level remains inert under ambient chemical conditions and will not ionize, form dative bonds, or hybridize with the next higher valence-active level(s):

**Table:** 

**Relation**	**Groups**	**Order of atomic one-electron energies**	**Comments**
(1)	0, 1	ε[(*n*−1)p] **≪**ε[*n*s] < ε[*n*p] **≪ε[(*****n*****-1)d]**	d-orbital collapse begins
(2)	2	ε[(n−1)p] **≪**ε[*n*s] < **ε[(*****n*****−1)d]** < ε[*n*p]	here the (*n*+ℓ, *n*) rule happens to apply
(3)	3–11	ε[(*n*−1)p] **≪ε[(*****n*****−1)d]** < ε[*n*s] < ε[*n*p]	element series of hardly varying ε values
(4)	12–18	ε[(*n*−1)p] < **ε[(*****n*****−1)d]** **≪**ε[*n*s] < ε[*n*p]	second step of d-orbital collapse

## Different Representations of Periodicity: Chemical Narratives

### From Correct Quantum Chemistry to Chemical Facts

*A large body of chemistry can be logically rationalized qualitatively, and theoretically simulated quantitatively, with the help of sufficiently digested quantum physics. Atoms and even more so molecules are rather complex systems with a pattern of observable properties that exhibits some coarse structure that is advantageous to be exploited in practice, with the help of fact-adapted Periodic Tables. For pedagogic introductions into the field of chemistry, ‘impressive’ and more simplistic (though yet pragmatically fact-adapted) designs may be most useful. Important points for chemistry are that chemically unbound neutral atoms in a vacuum may differ from chemically bound atoms in compounds, and that the energetic order of s,p vs. d,f valence orbitals changes after group 2 or 3, keeping the large orbital energy gap above closed noble-gas shells*.

Remarkable trends of chemical thought on several related issues in the present context emerged in the chemical community as accepted narratives during the past decades. Conceptually as well as in reality, the series of unperturbed neutral atoms in a vacuum can be obtained by stepwise adding a proton (and some neutrons) to the atomic nucleus, and simultaneously a ‘differentiating’ electron to the atomic shells. The leading electron configuration^8^ from which the physical ground *state* with the lowest energy *level* of the chemically non-bonded atom derives, depends in an involved manner on the Coulomb, exchange, and spin-coupling interactions of the many electrons in the atom (Condon and Shortley, 1935). Which leading orbital occupation scheme dominates in the ^*M*^$L_{J,i}^{P}$ ground level (*M* = spin multiplicity; *L* = total orbital angular momentum; *J* = total orbital + spin angular momentum; *P* = parity; *i* = parentage) depends in some cases on energy differences (Moore, 1949 et seq.) as small as thermal energies, while chemical bond interaction energies are up to several hundred times larger. Therefore, which electronic orbital configuration dominates in a free atomic ground states is a complicated issue (Schwarz, 2010b), the result being listed in the textbooks to train the memory. On the other hand, which configuration(s) dominate in chemically bonded atoms is a rather different issue, but plays a major role in chemistry; its understanding might be useful for chemists.

The simply structured physical laws of quantum mechanics, when applied to many-electron atoms (or even to chemical molecules) lead to a rather complicated set of results. Madelung (1936) mentioned an empirical finding that gave rise to an ‘idealized’ rule, how he called it, which reproduced the leading configurations of the outermost orbitals in the special field of *ground states of neutral free atoms* of all main-group elements, and of ca. 2/3 of the transition elements. A useful qualitative rule (here for vacuum spectroscopy of non-bonded atoms) should work, however, in at least 90 per cent of cases (Schultz, 2010).

When quantum mechanical concepts were absorbed by a broader chemical community in the middle of the twentieth century, a narrative evolved and was taken over by physicists, educators and philosophers, when they gave thought to the system of chemical elements. Namely, Madelung's (*n*+ℓ, *n*) rule was given a new interpretation (Scerri, 2007, 2020; Schwarz and Rich, 2010). Originally, atomic spectroscopists used the (*n*+ℓ, *n*) rule to memorize which ‘differentiating’ orbitals become additionally occupied in *free neutral atoms*, when element number *Z* increases stepwise. Chemical educators however applied it to the *chemically more interesting case of the various ions of a given element Z* with a stepwise increase of number of valence electrons. It is a pity that the (*n*+ℓ, *n*) rule usually fails when d and f orbitals are involved. Concerning the heavier p elements, the order of orbital energies corresponds to (*n*−1)d^10^-core < *n*s^2^ *n*p^g−12^, while the (*n*+ℓ, *n*) rule assumes the inverted order *n*s^2^ < (*n*−1)d^10^ *n*p^g−12^. Concerning the d elements, the (*n*+ℓ, *n*) rule fails to reproduce the leading^8^ configurations of the series of neutral free atoms in some cases, and of the series of oxidation states of a given d element in most cases.^9^

A further change of meaning was the interpretation of (*n*+ℓ, *n*) as reproducing a *universal energetic order of the atomic orbitals*. There are two aspects to such an interpretation. First, there is no universal energetic order. An important basic fact of the electronic structure of atoms is that the orbital energy order varies significantly with *Z* and also with the ionic charge [see relations (1–4); Figure 5; and footnote 9]. That was known, in principle, a century ago (Bohr and Coster, 1923). Further, an oversimplified ‘strict’ Aufbau rule (then called principle) is sometimes postulated that excludes the simultaneous occupation of energetically slightly different orbitals, something that is common in transition metal complex compounds of the weak ligand field type (Ballhausen, 1962).^10^

Eventually, the difference of *free atoms in space*, and of *bonded atoms in compounds*, was discarded. However, free atoms in vacuum have ample space around them. The diffuse *n*s Rydberg orbitals (see footnote 6) with weak e-e repulsion are energetically favorable in comparison to the compact (*n*−1)d orbitals. In molecules, however, the extended *n*s orbitals are energetically destabilized by Pauli repulsion of the occupied shells of the bonded ligand atoms (Wang et al., 2006), except for hydrides where the proton has no occupied core shells. A special case are the metals, where the crystal structure with high coordination numbers allows for delocalized valence bands, which support diffuse *n*s orbital occupation. A useful rule of thumb is that the leading configuration of a transition metal ion of oxidation state +*q, Z*^+*q*^, in group *g* of the periodic table, may be approximated by (*n*−1)d^*g*−*q*^ (Ballhausen, 1962; Jørgensen, 1969). The effectively neutral atoms in metals have the approximate configuration (*n*−1)d^g-1^*n*s^1^, while effectively negatively charged transition metal atoms in respective complexes have the leading (see foonote 8) configuration (*n*−1)d^g–1^*n*s^2^.

This common knowledge of transition metal chemistry has yet not entered the common chemical textbooks, which explicitly or implicitly teach that the electronic structure of unbound atoms in physical vacuum is an optimal paradigm for the electronic structure of bonded atoms in chemical compounds (Millikan, 1982; Schwarz, 2010b). In a famous article at the centenary of the Periodic Table, Löwdin (Löwdin, 1969) asked “the question at what degree of ionization the energy rule has become changed”. Since Madelung's rule holds for a significant fraction of the series of neutral free atoms, and the chemical (*n*+ℓ, *n*) rule for a small fraction of the series of differently charged ions of a given atom,^8^ any so-called proof in the more recent literature must appear problematic (e.g., Wong, 1979; Meek and Allen, 2002; Thyssen and Ceuleman, 2017; Kholodenko and Kauffman, 2019).

In their early searches for a periodic system, Meyer (Meyer, 1864, 1870) and Mendeleev (Mendeleev, 1869a,b; Mendelejeff, 1871) had cut the helical array of elements (Figure 3, right) at different places. In later years, the most common convention became cutting between the p-block halogens and the s-block alkali metals. It is there, where the largest variations of the pseudo-periodic chemical properties of elements occur. Examples of this convention are the ‘short’ table in Figure 1, and the nowadays more common ‘medium’ tables with 18 groups as in Figure 4 (with the f-block under the main table, a clever alternative to a ‘long’ table with 32 groups, printable on common paper format). Figure 4 was suggested (though not prescribed) by the IUPAC (the “Red Book” by: Connelly et al., 2005; IUPAC's archives: IUPAC, 2015).

Other options are the cut before or inside or after the d-block, so that the ‘pivots of periodicity,’ and the groups with dominant valence −1, 0, +1, show up somewhere in the middle of the table (Meyer, 1864; Mendeleev, 1869a). Before the discovery of the noble gases, there was no group with zero valence between −1 (halogens) and +1 (alkali metals); the void of chemical elements without any valence activity appeared natural and acceptable to the former chemists.

The (*n*+ℓ, *n*) rule assumes, for the values *n*+ℓ = 1 to 8 in the Periodic Table, a rather atypical orbital energy order, where the steps of *n*+ℓ are indicated by ‘≪’:

$$\begin{array}{l} \left(5\right)\;1s\ll 2s\ll 2p<3s\ll 3p<4s\ll 3d<4p<5s\ll \\ 4d<5p<6s\ll 4f<5d<6p<7s\ll 5f<6d<7p<8s \end{array}$$

**Figure 6:**
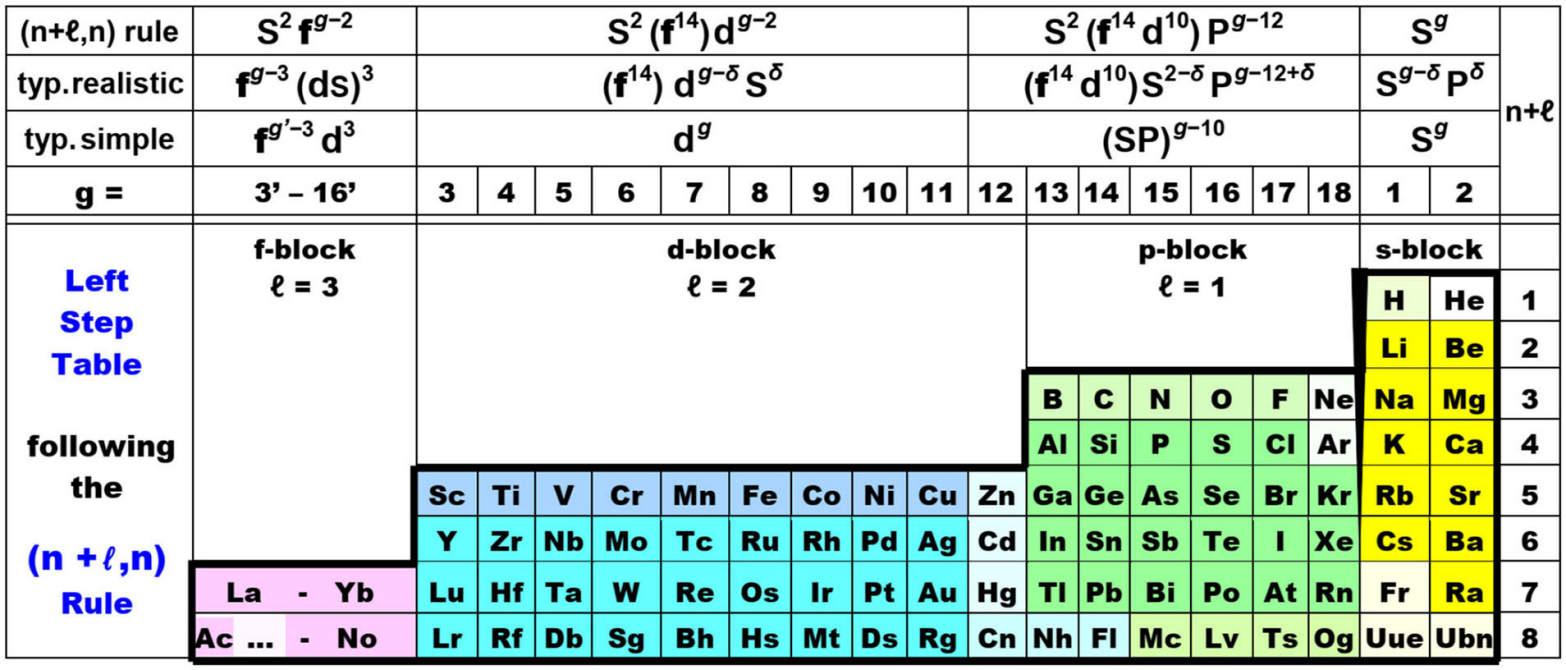
The elegant “Left Step” periodic table, following the canonized “(*n*+ℓ, *n*) rule,” see relation (5) below. −1st line at the top: Madelung's “idealized” occupations of the s, p, d, f (ℓ = 0, 1, 2, 3) valence shells in many *neutral unbound* free atoms in physical vacuum, taken over by the chemical community as the (*n*+ℓ, *n*) rule for less or more *polar-bonded* atoms in chemical compounds. −2nd line: typ. realistic = ℓ-occupations of typical, *bonded* atoms in real chemical compounds, where the case-dependent population parameter is mostly 0 < δ < 1. −3rd line: typ. simple = simplified approximate chemical valence configuration of typical bonded atoms. −4th line: **g** = group number = number of chemically active electrons in the valence shell of largely neutral atoms bonded at ambient conditions (modulo 10). —Colored block castes of elements: s—yellow, p—green, d—blue, f—lilac. Notes on variant backgrounds: —(a) Ac …: some early actinoids Pa to Pu (and possibly Th and Am) are chemically quite different from their lanthanoid counterparts (Ce)-Pr-Nd-Pm-Sm-(Eu). Conversely, Ac and the heavier actinoids Cm to Lr are quite similar to their lanthanoid counterparts La and Gd to Lu, and to Sc and Y. —(b) The 3d elements Sc to Cu are special in forming high- and low-spin, or weak- and strong-field, complexes with ligands from the left and right part, respectively, of the spectro-chemical series, while the 4d and 5d elements usually only form low-spin strong-field complexes with any ligands. —(c) Elements Zn-Hg of group 12 have closed d^10^ shells and are better regarded as members of the sp block. —(d) The 2p series B-F is unique with the 2s and 2p orbitals of comparable spatial extension and a strong tendency for sp hybridization. —(e) H and He are unique with having no extended atomic core and no other orbital energetically nearby 1s. —(f) He, Ne, Ar have well-closed 1s^2^, 2p^6^, 3p^6^ shells, respectively, at most forming complexes with weak secondary bonds, i.e., they have no 'real chemistry' under ambient conditions. In contrast, Be etc., and Kr etc. form primary bonds under ambient chemical conditions. —(g) The superheavy elements at the end of the bottom row feature peculiarities: Cn, Nh, Fl are predicted to have a chemically active d shell under ambient conditions. Fl, Mc, Ts, Og have comparably inert 7s½^2^ (and 7p½^2^) pairs. —(h) Fr, Og, Uue, Ubn are predicted to have unusual valence-active (*n*−1)p^3^/${\;}_{2}^{4}$*n*s^0−2^ shells, i.e., no closed p^6^ noble-gas shell.

Relation (5) maps onto the periodic table design of Figure 6, with 8 periods up to Z = 120, being called the Janet *Left Step Periodic Table* (LSPT; Janet, 1930; Scerri, 2007, 2020; Stewart, 2010, 2020). The LSPT looks particularly ‘elegant’ and ‘symmetric’ with regularly arranged s, p, d, f blocks. The LSPT is obtained by cutting the periodic spiral in the left middle of the sp block, i.e., after the open (sp)^2^ shells, and then shifting hydrogen and helium (with closed 1s^2^ shell) above lithium and beryllium. The simple outer shape of the LSPT may be useful in introductory chemistry courses (Kurushkin, 2017). While the assumed systematic valence electron configurations of the rule (1st line in Figure 6) differ quite a bit w.r.t. to orbital order and orbital occupation from *real chemically bonded atoms* (2nd line; see also **Figure 8**), the graphic nevertheless appears useful to display the chemical trends. However, it must be noted that for the heavy p-block elements, the (*n*+ℓ, *n*) rule shifts the occupied (*n*−2)f^14^ and (*n*−1)d^10^ shells in between the *n*s^2−δ^ and *n*p^g−12+δ^ valence shells, while the observed energetic order is (*n*−2)f ≪ (*n*−1)d ≪ *n*s < *n*p. Concerning the d-block, in the vast majority of cases there is no *n*s^2^ shell below the (*n*−1)d^g−2^ shell, but a weakly occupied *n*s shell somewhat above (except for negatively charged transition metal atoms with approximate *n*s^2^ occupation, and for metallic phases with neutral metal atoms and approximate *n*s^1^ occupation).

The zinc-group 12 is also special (Jensen, 2003), since d^10^ is no longer valence-active, but behaves as an inert core shell. These “d-block” elements behave chemically as typical s-block members. The yet unconfirmed case of mercury tetrafluoride (HgF_4_) is no counter-argument, since it is labile under standard conditions (Wang et al., 2007; Jensen, 2008; Rooms et al., 2008; Ghosh and Conradie, 2016; Gao et al., 2019; Lin et al., 2020). Finally, for the f-block, the dominant valence-bonding shell is (*n–*1)d; the (*n–*2)f falls deeper into the atomic core, the higher the effective positive charge on the atom becomes; as a rule of thumb, the f contributes to covalent bonding only in some cases of Ce(4f5d) and for Pa to Pu (5f6d), while the *n*s shell only weakly contributes to bonding, as in the other d-elements.

Various clarifying comments are necessary (legend of Figure 6) when looking from the viewpoint of the (*n*+ℓ, *n*) rule onto the behavior of *real chemical elements*. Admittedly, this holds partially also for other designs of Periodic Tables (such as in Figures 1–5). To explain the chemistry of the elements, one must consider not only the correct leading (see foonote 8) valence configuration, but also the correct configuration of the occupied core shells, and not only the orbital energies but also the orbital radii. For instance, the valance *n*s orbitals are very diffuse (see footnote 6) at the beginning of a period (or at the very end of the LSPT) so that they play little role for covalent overlap interactions. The positioning of a closed-shell element (such as He-1s^2^) above an open-shell (such as Be-2(sp)^2^) has no basis in chemistry or quantum mechanics.

### From Partial Aspects of Reality to Created Patterns

*The chemical trends along the Z-line of elements are non-linear and of different characters. The regular grid of periodic tables is adapted to the wish for a well-ordered presentation, but curtails the chemical facts. The common IUPAC Table is already half-way between realistic aim-dependent presentations of some details of the natural System of Elements and the idealization of a desired overall appearance of a symbol for the System in the form of a Periodic Table*.

We had noted the two points before that different chemical behaviors are relevant in *different contexts* such as under ‘common’ or astrochemical or geochemical conditions, and that some chemists prefer to classify *borderline cases* in either this or that rigorous, unique manner. Combined with the *fuzzy nature of chemistry* (Syropoulos, 2020), this may lead to futile disputes, including those over the Periodic Table (Schwerdtfeger et al., 2020). Chemistry has all sorts of fuzzy definitions such as chemical periodicity or chemical bonding or hydrogen bonding etc. Rather than a black or white categorization, the IUPAC definition of a *hydrogen bond* (Arunan et al., 2011) suggests the strategy that classification is more reliable and less open to controversy, the greater the number of given criteria is satisfied. The range of *metalloids* on the metal to non-metal divide (Figure 1, right; Vernon, 2013) or the representation of *groups 3 and 13* including the f-block are further examples. Such issues are less disputed in practical chemistry.

Several theoretically oriented chemists argue that chemistry loses its basic ingredients (i.e., techniques useful to handle a complex field) and becomes like physics (which is adapted to handle the simple basic structures of reality). Some argue in favor of different practices, while others argue against any fuzzy concepts including those that have proven useful in previous times for classifying the nearly continuous distribution of observations, with few borderline cases remaining. However, some scholars do not like ambiguous borderline cases. It is our experience that many young students expect that a teacher should have a simple answer to any (complicated) problem.

No such loss of chemical richness is warranted. Jones (2010) cogently summarized the situation: “Scientists need not lose sleep over the hard cases. As long as a classification system is beneficial to *economy of description*, to *structuring knowledge* [italics added] and to our understanding, and hard cases constitute a small minority, then keep it. If the system becomes less than useful, then scrap it and replace it with a system based on different shared characteristics.” In the case of hydrogen and helium, for example, we agree with the suggestion of Schwerdtfeger et al. (2020): “Although hydrogen and helium are clearly separate from the rest of the PTE, almost every chemist agrees that we can leave these elements in their current place in the PTE, *keeping their distinctive quantum nature in mind* [italics added].”

In another sense, the problematic cases can be regarded as addressing philosophical issues that border on what a periodic table tries to represent. What you get from your Periodic Table is what you put in, unlike the nature-given Periodic System. The learning is to consider how many insights and how much understanding could be gained from appreciating these different stepping stones including, but not limited to, the fundamental and important nature of inanimate matter. The takeaway is to explain some relevant context to readers, colleagues, and students.

- The so-called IUPAC Table (Figure 4) is more of a chemistry-focused Pragmatic Table.
- The form with lutetium instead of lanthanum in group 3 is more of an Idealized Table, instead of a Pragmatic Table with “no need to lose sleep” (Scerri, 2020).
- In a Solid-State Physicist's Table, both lanthanum and lutetium, as 5d metals, go under yttrium (Vosko and Chevary, 1993).
- In the electronegativity-focused Pauling Table (Pauling, 1953), group 3 is boron, aluminum, scandium, yttrium, lanthanum, and actinium.
- Aluminum over scandium is more of a Metallurgist's Table (Habashi, 2009).
- Geochemical Tables (McSween and Huss, 2010; Railsback, 2018) emphasize property trends important for the earth scientist, i.e., they give up the beauty of symmetrized arrangements in favor of irregular chemical facts. Some tables define the carbon-silicon group as containing titanium, zirconium, hafnium rather than the standard set of germanium, tin, lead.
- In the Astronomer's Tables (Esteban et al., 2004; McSween and Huss, 2010; Yamamoto, 2017), hydrogen and helium are the only non-metals and all the other elements are labeled as metals.
- In a Superconductivity Periodic Table, group 2 is split into: barium and radium; calcium, strontium, and ytterbium; group 12 is beryllium, magnesium, zinc, cadmium, and mercury (Wittig, 1973).
- A periodic table with hydrogen over boron makes for a nice Designer Table (Luchinskii and Trifonov, 1981).

The basis of element grouping may be a (sometimes unspecified) selection of facts, or a preset and appealing pattern useful in education or promotion.

## Atypical Periodicities

### At Top and Bottom

*The chemistry of elements is richer than being satisfactorily pictured in a table. The individuality of elements from the same similarity group is most pronounced at the top, where the number of valence orbitals is small (one 1s, four 2sp) and the orbital energies and radii form bands with gaps. At the bottom of the periodic table, these distinctions become washed out. That is due to both the non-relativistic increase of the density of states and different screening effects, and the relativistic orbital shifts and splittings energy- and radii-wise*.

Already Gmelin (Gmelin, 1843) and Mendeleev (Mendeleev, 1869a,b; Mendelejeff, 1871) had noted that the lightest elements of the similarity groups exhibit somewhat peculiar chemical behaviors. Mendeleev labeled the elements of the second period the typical ones (типические элементы, sometimes translated as representative elements), and we today call groups 13, 14 etc. the boron, carbon etc. groups. Biron (Biron, 1915) recognized a zig-zag behavior down the main-group elements with similar extrema in periods 2, 4, and 6, and called it secondary periodicity. Jørgensen (Jørgensen, 1969) and Shchukarev (Shchukarev, 1977) discussed the peculiarity of the first element of any group of the periodic system in great detail and related it to the comparatively *small radii of orbitals without radial nodes*: 1s (hydrogen), 2p (boron to fluorine), 3d (scandium to copper), and 4f (cerium et seq.). Kutzelnigg (Kutzelnigg, 1984) explained the special behavior of the light 2p main-group elements as due to the similar extensions of the 2s and 2p valence orbitals, which supports sp hybridization. In contrast, the p-valence shells of the heavier p-block elements are significantly more extended than the respective s-valence shells, which is beneficial for pure σ(p) bond formation. Shchukarev (Shchukarev, 1977) in the ‘East’ named the feature of the radial nodelessness of the (*n*,ℓ=*n*-1) valence orbitals *kaino-symmetric* (kainos = new), while Pyykkö (Pyykkö, 1979a,b) in the “West” introduced the term *primo-genic* (primus = first). The basic relevance of the radii of the atomic valence orbitals was later discussed in review articles (e.g., Kaupp, 2006), but hardly entered the chemical textbook scene (an example of where it did: Huheey et al., 2014).

When *Z* increases, kainosymmetric orbitals with new primogenic ℓ-values become occupied above the noble-gas core shells [1s^2^ and 1s^2^-(*n–*1)p^6^] at energies ε_nℓ_ = – (*Z*_eff_/*n*_eff_)^2^. At the beginning of period *n* with just a few valence electrons, the value of *n*_eff_ can be modeled by *n*_eff_ = [*n* – δ_screen_ + ℓ(ℓ+1)/6], where δ_screen_ is the screening of the nuclear attraction of the *n*s valence shell by the noble-gas core. For ℓ = 0 and 1, i.e., ℓ(ℓ+1)/6 = 0 and 1/3, the valence s and p shells appear nearly together after the noble-gas shell closure. d and in particular f with ℓ = 2 and 3 appear later corresponding to ℓ(ℓ+1)/6 = 1 and 2, meaning that (*n***−2**)f and (*n***–1**)d appear nearly together with (*n***–0**) (s,p). Because of the quantum constraint ℓ ≤ *n*–**1**, new electronic shells in screened atomic Coulomb potentials appear in double-steps. Therefore, there are two periods each of length 8 for 2sp and 3sp, of length 18 for 3d4sp and 4d5sp, and of length 32 for 4f5d6sp and 5f6d7sp. Apparently, there is no physical-chemical reason for two *n*s periods before the first (sp)^8^ period, except the desire for more symmetry and beauty in the generated Periodic Table (Jensen, 1986). The appearance of the kainosymmetric 2p, 3d, and 4f shells every second period causes the secondary periodicity with the scandoid and lanthanoid contractions of the effective atomic bond radii. Many parameters of the elements and their atoms derived from the individual chemical observations can be approximated as expansions in terms of the number of ℓ-valence electrons (Imyanitov, 2019a,b) and in terms of 1/*Z* = *Z*^−1^ (Layzer, 1959), at least within non-relativistic quantum chemistry.

However, the real world behaves quantum-relativistically. The errors of the non-relativistic approximation are conventionally called the “relativistic corrections,” which can be expressed by expansions in terms of powers of *Z*^2^ (e.g., Schwarz, 2010a). Consequently, it is difficult to make reliable predictions on the chemistry of the heavy elements with high *Z*, i.e., with increasingly larger terms of *Z*^2^, *Z*^4^, etc., by *extra*polation from the region of the lighter “non-relativistic” elements. In contrast, *inter*polations within the region of the lighter half of the elements, say in the first five periods up to *Z* = 54 (xenon), are easily successful, as Mendeleev had demonstrated. Below we will draw attention to some basic though empirically un-expectable chemical phenomena of the heavy elements in the 7th period, on the basis of the few chemical observations and quantum-chemical calculations (e.g., Nash, 2005; Pyykkö, 2012a,b; Pershina, 2015; Schädel, 2015; Türler et al., 2015; Türler, 2016; Düllmann, 2017; Giuliani et al., 2019; Trombach et al., 2019).

The chemically most relevant trends due to the ‘relativistic corrections’ are (Schwarz, 2010a; Pyykkö, 2012a):

- The *n*s-levels are energetically stabilized and spatially contracted, with the (*n–*1)d^5^/_2_ – *n*s½ and (*n*−1)p^3^/_2_ – *n*s½ gaps being reduced.
- The p-levels are also stabilized and contracted, and strongly spin-orbit-split, so that the *n*p½ spinor level is also contracted and stabilized toward the *n*s½ level; but the sp hybridization is hampered because of the complex structure of the p½ spinor.^4^
- The p^3^/_2_ valence shell is destabilized, therefore the p½ – p^3^/_2_ gap is increased and the gap between the p^3^/_2_ to the next s½ is decreased.
- Due to the orbital angular momentum of quantum numbers ℓ= 2 and 3, there emerges a significant centrifugal force ~ℓ(ℓ + 1)/2*r*^3^. Therefore, the d and f orbitals do not strongly penetrate the inner atomic core shells and are better shielded from the nuclear attraction due to the relativistic sp contraction of the s and p type shells. There results an ‘indirect’ destabilization of d and f shells, whereby the (*n*−1)d^5^/_2_ – *n*s½ gap is further reduced (see also above).

### Orbital Energies and Radii at the Bottom

*At the bottom of the periodic table, relativistic orbital changes become qualitatively relevant for chemical thermodynamics. The gap between the (n*−*1)p*^3^*/*_2_ *noble-gas core shell and the s-metallic valence shell decreases. In the early actinoid series, 5f and 6d can hybridize. The (n*+ℓ*,n) rule may hold for the first time in the 6d series. The level pattern of 6d*^3^*/*_2_ –* 6d*^5^*/*_2_ –* 7s*½ –* 7p*½ –* 7p*^3^*/*_2_ –* 8s*½ *at the middle and end of the 7th period changes qualitatively, suggesting a different chemistry and change of periodicity at the end of the 7th period and the start of the hypothetical but practically meaningless 8th period*.

Because of the large relativistic spin-orbit coupling in the heavy elements, it becomes mandatory to consider the spin-orbit coupled spinor-orbitals (see footnote 5). Only for lighter elements, the picture of space-orbitals with different spins is an acceptable approximation. It is sufficient for instance for the comparatively weak spin-orbit induced “heavy-atom” corrections to “spin-forbidden” transitions in spectroscopy and in kinetics. Every element has a different core and a different valence shell (Figure 12 of: Cao et al., 2019), which together determine the chemical behavior in a physically lawful, though effectively rather complex manner. A general understanding of the system of chemical elements can be obtained by an analysis of the trends of the energies and radii of the outer-core and valence shells. The inner core and the outer Rydberg shells, which are important in XUV and UV spectroscopies, are less relevant for genuine chemistry and will not be considered here.

**Figure 7:**
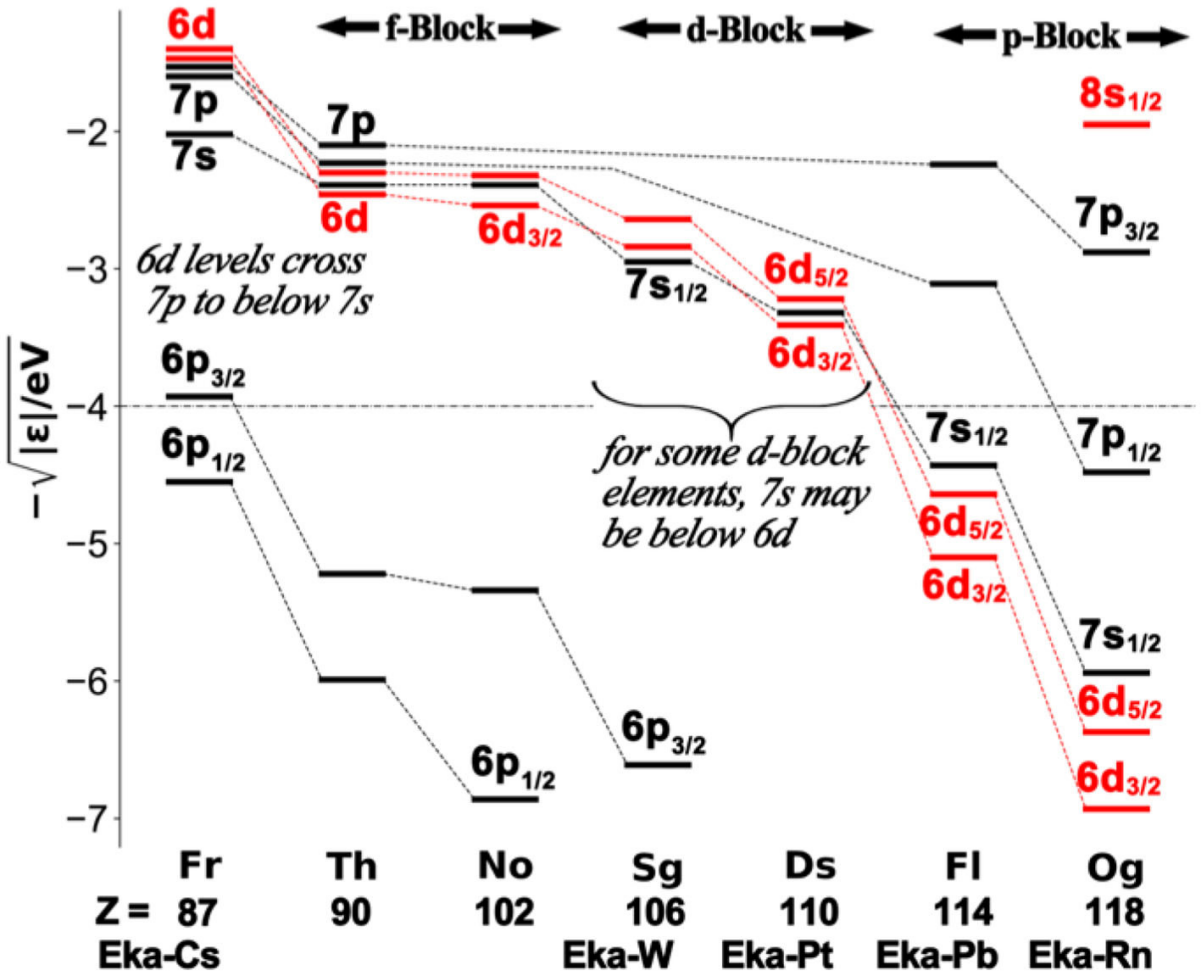
Atomic one-electron energies of the outer-core and the valence shells, displayed as –√**|ε**/eV**|**, over the lowest period 7 of the system of elements from francium to oganesson, at the Dirac-Fock level of approximation.

In Figure 7, we display the energy levels ε of selected atoms of period 7 from ‘alkali metal’ francium (_87_Fr, group 1) to ‘noble gas’ oganesson (group18) (see footnote 4), with two representative elements for each block, namely _90_Th and _102_No (Eka-Yb) for the f-block, _106_Sg and _110_Ds (Eka-Pt) for the d-block, and _114_Fl (Eka-Pb) and _118_Og for the p-block. In order to show both weakly and strongly bound shells in the same graphic, we apply a square-root scale γ = −√|ε/eV| ~ Z_eff_/n_eff_. The horizontal dashed line at γ = −4 is near to the value of the O^0^-2p shell. Electronegative ligands such as oxygen, fluorine or chlorine would form homopolar bonds with atomic shells having γ ≈ −4 [provided the overlap conditions are favorable and the number of valence electrons does not require filling the antibonding companion level(s)]. More or less electronegative ligands will lead to polarized covalences, where the charge transfer is partially counter-balanced by lowering/raising the ε values of the positively/negatively charged atoms, respectively.

**Figure 8:**
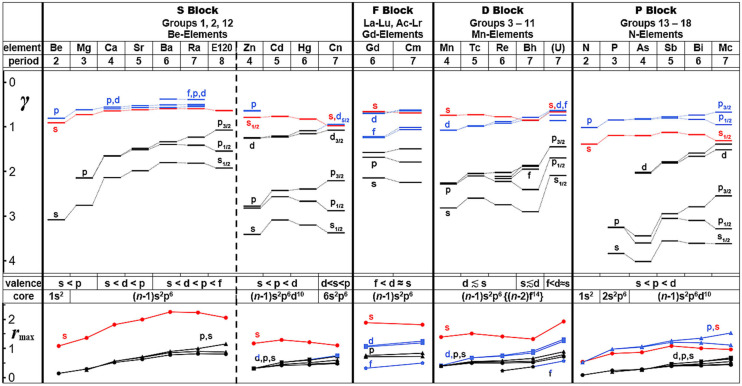
Energies and radii of atomic shells of main and transition group elements, governing their chemistries (representative, light to heavy members, from the s and p blocks, and from the d and f blocks) from atomic Dirac-Fock computations. *Upper part:* Energetic order of the chemical valence shells (top: s in red; p, d, f in blue) and of the outer core shells (bottom: in black) in terms of parameter **γ** = √|ε/Ry|= Z_eff_/n_eff_, where ε is the orbital energy, corresponding to the experimental single-electronic ionization energy (in units of Rydberg Ry = 13.6 eV or 1313 kJ/mol). *Lower part:* Radial density maxima ***r***_max_ of the atomic shells, in Å (color code of valence and core shells as above).

In Figure 8, both the one-electron orbital energies and radial-density-maxima of the outer-core and the valence shells are shown, for atoms down a representative group of the s-block (group 2; for comparison the closely related group 12 is also displayed), the f-block [the central f^7^(ds)^3^ elements]; the d-block (middle group 7); and the p_1/2, 3/2_ block (middle group 15). The devised states in a model of independent electrons in the mean field of a many-particle atom or molecule are characterized by the positional and spin distributions in three-dimensional space. The spin-orbit coupling for states of spatial angular momentum ℓ in a central field causes energy and radial changes approximately proportional to *c*_ℓ_·ℓ(ℓ+1). The spin-orbit splitting increases quadratically with angular momentum ℓ, but the prefactor *c*_ℓ_ typically varies as ℓ^−3^, because the radial spin-orbit coupling strength decreases with ℓ because increasing ℓ keeps the electron away from the atomic center where the coupling is largest. Consequently, in a given energy-shell of the atom, the spin-orbit effect is counter-intuitively the larger the smaller the orbital angular momentum is, that is, largest for the p-shell. The common space-orbital model with p_x_, p_y_, and p_z_ is no longer qualitatively correct for the “super-heavy elements” (SHE), but must be replaced by the spinor-orbital model. In Figure 8, the spin-orbit splittings of the energies (γ ~ √-ε) and radii (***r***_max_) of the p-shells – both the outer-core and valence shells – are formidable for all heavy atoms, while the d and f splittings are less pronounced.

Now, which bonding patterns of the elements of the 7th period may be expected against this background? In the following subsections, we will individually discuss the heavier members of: the s-block (groups 1–2); the early d-block including the f elements (groups 3–5); the later d-block (groups 6–11); the s½ p½ block (groups 12–14); and those of the p_3/2_ block (groups 15–18).

## The Blocks

### The s-Block Elements

*The heaviest elements of groups 1 and 2*, _87_*Fr*, _119_*Uue, and*_120_*Ubn, are predicted as similar to the middle s-block elements. However, they may behave in a Janus-faced manner depending on the conditions, i.e., like typical low-valent s-block elements or very differently like penta- and hexa-valent heavy p-block elements*.

Upon increasing the element number of a noble gas with outer closed shell 1s^2^, 2p^6^, 3p^6^, …, the additional electrons in the respective alkali and alkaline earth metals of periods *n* = 2, 3, 4, … are accommodated in the rather *diffuse* (*n*sp)^2^ valence shell (with the option of some (*n*-1)d admixture from period *n* = 4 onward (Woolman et al., 2018; Li et al., 2019; Fromm, 2020). The s-block elements appear strictly mono- and di-valent under common conditions. While the ionic compounds have a formally empty valence shell, the partially covalent complexes and organometallic compounds as well as the metallic phases exhibit non-negligible s-p(d) occupation, and orbital mixing due to the small *n*s-*n*p or *n*s-*n*p-(*n*-1)d energy gaps. In particular, Be and Mg are *n*s(p) valence-active, while Ca, Sr and Ba are *n*s(*n*-1)d valence active (Fernández et al., 2020).

From the quantum-theoretical as well as from the chemical-empirical points of view, there is an objective qualitative difference between the alkaline-earth-metal and helium atoms, since He has a rather compact, closed (1s)^2^ shell (without any appreciable p admixture). Helium remains zero-valent even under pressure, such as in the cubic inclusion compounds [${\text{Na}}_{2}^{+}$ (e_2_)^2−^ He^0^] or [${\text{Na}}_{2}^{+}$ (O)^2−^ He^0^], where formally He(1s^2^)^0^ and (1s^2^)^2−^ or O(2p^6^)^2−^ respectively are inserted into the voids between the Na^+^ ions of a new pressure-induced structure of Na (Dong et al., 2015, 2017; Rahaman et al., 2018; Zou et al., 2020). Depending on the chosen partitioning of the atoms in molecules and crystals, the obtained effective charges are Na^+(1−δ)^, (e_2_ or O)^−(2−η)^ and He^(2δ−η)^ with small numbers δ and η. For (2δ−η) > or < 0, narratively oriented chemists may postulate bonding attractions of the chemically inactive He, either of ionic He^+^-O^−^ or He^−^-Na^+^, or of covalent He^+γ^ → O^−γ^ or He^+γ^→*Na*^−γ^ type. This is a nice example of the different views within and between the Two Cultures.^11^

Figure 8 shows just a little variation of the s valence shell energies and radii for the s-block members, with flat extrema at period **6**, of ionization potential, electron affinity, electronegativity and effective atomic radii. Elements _119_Uue (Eka-Fr) and _120_Ubn (Eka-Ra) in period **8** were accordingly predicted to resemble the lighter homologs in period **4** (Türler and Pershina, 2013; Pershina, 2015; Chemey and Albrecht-Schmitt, 2019). One must consider however that the *n*s shells of these heavier elements, in particular of the heavier alkali metals, are spatially very diffuse, yielding only weak overlap interactions with small differences among each other, but differing from the group 12 elements.

For the heaviest elements, however, the highest core level (*n*−1)${\text{p}}_{3/2}^{4}$ moves up into an energy range typical for strongly electronegative elements, and becomes radially less compact (Figures 7, 8). Fricke and Waber (Fricke and Waber, 1971; Fricke, 1975) had already speculated about raised valences of elements _119_Uue and _120_Ubn. In more recent years, computations and experiments up to the megabar range have established the stability of alkali and alkaline-earth polyhalides under high pressure met inside the planets (Dong et al., 2015, 2017; Zhu et al., 2015; Goesten et al., 2017; Miao et al., 2017; Luo et al., 2018; Lin et al., 2019; Rahm et al., 2019). Under standard conditions polyfluorides CsF_n_ and BaF_n_ are at most metastable, with a decay barrier that may render their temporary stability possible under standard pressure only at exotically low temperatures (see e.g., Rogachev et al., 2015; Vent-Schmidt et al., 2015). This also holds for HgF_4_ (Wang et al., 2007; Jensen, 2008; Rooms et al., 2008; Ghosh and Conradie, 2016; Gao et al., 2019; Lin et al., 2020).

Apparently, cesium, barium and mercury from period 6 are borderline cases, and it may well be that the heavier homologs francium, radium, (_112_Copernicium), and _119_Uue, _120_Ubn, behave no longer as typical alkali and alkaline-earth elements but form higher-valent complexes similar to those of the late heavy p-block elements such as [SbF_6_]^−^ or TeF_6_, stable in ambient conditions. Explicit molecular calculations by Cao et al. (2019) indicate thermodynamic *stability* of [Fr(V) F_6_]^−^, [Uue(V) F_6_]^−^ and [Ubn(VI) F_6_]^0^ under *standard* conditions against loss of F_2_, for instance

(6) [Uue(V) F_6_]^−^ ↛ [Uue(IV) F_5_]^−^ + ½ F_2_ ↛
[Uue(III) F_4_]^−^ + F_2_ ↛ ….

As indicated in Figure 1, right, the (*n*−1)p^6^ shell is chemically inert under ambient conditions in periods *n* = 3 and 4 from group-0 elements neon and argon onward. But (*n*−1)p^6^ is still chemically active in krypton and xenon. (*n*−1)p^6^ becomes an inactive noble-gas core shell in periods *n* = 5 and 6 only from group 1 elements rubidium and cesium onward. The (*n*−1)p^6^ shell becomes inert in period *n* = 7 from group 2 element radium onward, while in period *n* = 8 it is apparently active even in group 2 element _120_Ubn.

The theoretically predicted unexpected behavior of francium, _119_Uue and _120_Ubn, i.e., being poly-valent and forming polyhalide complexes, has hardly any direct practical-chemical consequences. The lifetimes of all francium isotopes are shorter than 1/3 h. In the real lab, only single francium compound molecules in a beam in vacuum, or on a surface, or in a matrix, or in chemically related compounds doped with tracer amounts of francium, could be investigated by ‘quick’ researchers. _119_Uue and _120_Ubn are the next elements to be synthetized in the coming decades and are expected with lifetimes far below a ms. The chemistry of possible compounds of such chemically ‘non-existing’ elements is yet relevant as they form reference points for the varying chemical trends between the upper and lower ends of any group in the chemically finite Periodic Table.

In summary, the ‘noble gas core shell’ is inert under ambient conditions for the three light noble gases helium, neon, (argon), but chemically active for the three heavier congeners krypton, xenon, radon, and also for oganesson being predicted a semi-metallic semi-conductor (Mewes et al., 2019a); with an expected bandwidth of 1.5 eV, Og could even have a metallic appearance. Of course this would only be true if the short-lived nuclei would live very much longer.

### The Heavy Early d-Block Elements Including the f-Block

*The two sets of group-3 to group-4 elements, lanthanum to hafnium, and actinium to rutherfordium, are typical early (n*−*1)d-elements with a little admixture of ns. From cerium (group 4') to ytterbium (group 2'), and from protactinium (group 5') to nobelium (group 2'), some valence electrons can be variably stored in the “f-cellar” of the atomic cores. In addition 4f contributes to chemical bonding for cerium (and praseodymium) and 5f contributes to bonding for protactinium to plutonium (and americium, groups 8' and 9')*.

The most important theoretical aspect here is the non-uniform spatial contraction and energetic stabilization of the 4f5d shells vs. the 6sp shells, and of 5f6d vs. 7sp, as functions of the element number *Z* and the effective charge *q* of the atom in a compound: ε(*Z, q*) and *r*(*Z, q*). The ‘comparatively simple’ ε(*Z*) behavior of 4sp vs. 3d was sketched in Figure 5. In the first two groups, the (*n*−1)d and (*n*−2)f shells are high-energy, diffuse Rydberg levels, hardly contributing to bonding in the majority of cases (Ji et al., 2015; see however: Levy and Hargittai, 2000; Wu et al., 2018a,b), which changes from group 3 onward.

How to define the f-block (i.e., including, or not, lanthanum and actinium or lutetium and lawrencium, or both pairs) within the series of early d-block elements is a still ongoing, standpoint-oriented controversy of philosophical, though of little chemical relevance (Edelstein et al., 2010; Morss et al., 2010; Pyykkö, 2019; Vernon, 2020b). Covalent contributions to the bonds of all these elements are dominantly based on the (*n*−1)d orbital overlap interactions. In period 6, the energy and radius of the 4f shell lends itself to additional covalent bond contribution in Ce(III) and in a few praseodymium compounds (Dolg and Moossen, 2015; Moossen and Dolg, 2016; Zhang et al., 2016; Hu et al., 2017; Smiles et al., 2020). Elsewise, the 4f level is too contracted, sitting inside the atomic core with outer (*n*−1)p^6^ shell (Figure 8), but can store electrons thereby changing the oxidation state, the number of d-valence electrons and the ionic core radius (Dognon, 2014, 2017; Liu et al., 2017; Pathak et al., 2017; Lu et al., 2019). In particular, the bond-oriented σ and π components of the f-shell can better contribute to overlap-binding, while the δ and ϕ components remain more contracted. The *n*s orbital is still dominantly Rydberg-like, see ***r***_max_ in Figure 8.

Due to the radial node effect (meaning the correlation of small radius and no radial node of atomic orbitals, reviewed by Kaupp: Kaupp, 2006; Huheey et al., 2014; Wang et al., 2020), the 5f contraction along the series of elements occurs more slowly than 4f, so that f still contributes to the covalence of protactinium, uranium, neptunium and plutonium, and in special cases of thorium (potentially) and americium, too, as a rule of thumb (Morss et al., 2010; Neidig et al., 2013; Ortu et al., 2016; Liu et al., 2017; Vitova et al., 2017; Wilson et al., 2018). From the common chemical empirical point of view, the elements thorium, protactinium, uranium, neptunium, and plutonium are more akin to the lighter outer transition elements hafnium, tantalum, tungsten, rhenium, and osmium, than to their officially homologous inner transition elements cerium, praseodymium, neodymium, promethium, and samarium. Indeed, during the first century of periodic tables, i.e., until Glen Seaborg (Seaborg, 1946), the early actinoids resided in the d-block.

Also at the second beginning of the series of 5d and 6d elements (lutetium and lawrencium), the (*n*−1)d and *n*s orbitals play the dominant role. An interesting example of 6d-chemistry is lawrencium (Xu and Pyykkö, 2016). Despite the recent excitement that the spin-orbit coupled ground state of the chemically unbound free lawrencium atom is of p-type, ^2^${\text{P}}_{1/2}^{\text{o}}$ (5f^14^6d^0^7s^2^7p^1^), the chemistries of Lu and Lr are found to be of typical f^14^-contracted d(s) type.

### The Later d-Block Elements

*The d-elements of periods 6 and 7 appear rather similar at first glance. However, the 6d*^5^*/*_2_ *shell becomes relativistically scalar and spin-orbit destabilized and expanded, while the 7s*½ *shell is stabilized and contracted so that the (n*+ℓ*,n) rule of the chemical textbooks appears to hold for the first time in the d-block. This causes various changes in the bonding details. The 3d and 6d series differ from the pair of 4d and 5d series*.

The di-metallic molecules M_2_ in periods 4, 5, and 6 have a comparatively large number of bonding orbitals derived from the ligand-overlapping (*n*−1)d-shells. The *n*s orbitals play only a little role except when the electropositive metal atoms carry small or negative changes. As an example, the di-tungsten molecule may be symbolized by Lewis formula |*W*
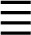
*W*| and leading electron configuration ($\sigma_{\text{d}}^{2}$ $\sigma_{\text{s}}^{2}$ $\pi_{\text{d}}^{4}$ $\delta_{\text{d}}^{4}$), with four bonding molecular orbitals σ_d_, σ_s_, and π_d_, and hardly any bonding orbitals δ_d_ (Many authors in the literature count the basically non-bonding δ orbitals as bonding, to get a higher bond order, see: Roos et al., 2007; Ruiperez et al., 2011; Li Manni et al., 2012; Sun et al., 2013; Singh et al., 2016; Chen et al., 2017). In the heaviest dimers, however, because of the (*n*−1)d-*n*s inversion in period 7 (Figures 7, 8), some (*n*−1)d-type molecular orbital is replaced by an *n*s-type orbital. As a result, the di-seaborgium molecule is to be represented by Lewis formula |Sg≡Sg| ↔ |Sg≡Sg| with leading electron configuration ($\sigma_{\text{s}}^{2}$ $\pi_{\text{d}}^{4}$ $\sigma_{\text{d}}^{2}$ $\sigma_{\text{s}}^{*}2$ $\delta_{\text{d}3/2}^{2}$), where the non-bonding $\delta_{\text{d}5/2}^{2}$ pair is replaced by the antibonding molecular orbital $\sigma_{\text{s}}^{*}$. Thereby the bond order (now only three) and the vibrational force constant are reduced and the bond length is increased. Similar changes occur for all dimers of periods 6 and 7 from groups 4 to 8 (Wang et al., 2016).

In the case of the coinage metal dimers M_2_ from group 11 (Cu_2_, Ag_2_, Au_2_), the M–M single bond is due to interaction of σ_(n−1)*d, ns*_ valence-hybrids between the polarized M-(*n*−1)d^10^ closed shell cores. In Rg_2_ however, the upper antibonding $\sigma_{\text{u}-6\text{d}}^{*}2$ orbital has changed its character due to the relativistic 6d^5^/_2_ destabilization and 7s½ stabilization to dominantly $\sigma_{\text{u},7\text{s}}^{*}2$ type, with remarkable changes of force constant, bond energy and charge and pair density distributions (Li et al., 2018).

Increased relevance of the *n*s valence orbital has also been verified in quantum calculations of various complexes of the period-7 transition elements such as [MO_4_]^0, q−^ and its thio- analogs [MS_4_]^0, q−^ (Huang et al., 2016, 2017; Hu et al., 2018). At the end of the d-series, the 6d_5/2_ shell is still energetically high enough to supply all electrons to form Rg-F bonds in Rg^7+^(6${\text{d}}_{3/2}^{4}$ 6${\text{d}}_{5/2}^{0}$ 7${\text{s}}_{1/2}^{0}$)F_7_ while stoichiometric AuF_7_ is still Au^5+^F_5_·F_2_ (Himmel and Riedel, 2007; Conradie and Ghosh, 2019).

### The Early p-Block Elements, Including Group 12

*The d-shell becomes chemically inert from group 12 onward. Only under exotic conditions can the d*^10^ *shell of mercury be oxidized, while this is predicted as easily possible in period 7 for copernicium, and possibly for nihonium. On the other hand, the 6s and 6p1/2 shells are remarkably stable for mercury, thallium and lead, and even more so for the heavier homologs, leading to an inert 6d*^10^*7s*^2^ *core for flerovium*.

As in the case of the s-block, scholars usually focus on aspects known from the upper part of the periodic table, that is, here on the *n*s and *n*p valence shells. Then, for the heavy members, only the relativistic stabilization and contraction of the *n*s_1/2_ and *n*p_1/2_ shells (Figures 7, 8) are discussed, whilst the destabilization and expansion of the upper (*n*−1)d_5/2_ core shell is rarely considered. Zinc, cadmium and mercury, with beryllium and magnesium as their precursors, are divalent throughout, which also holds for the XM–MX compounds of beryllium (I) through mercury (I). On the other hand, higher valent species have sometimes been reported such as apparent zinc (III) complexes with a broken 3d^10^ shell, but were not generally accepted (Schlöder et al., 2012).

However, mercury (IV) compounds with broken 5d^10^ shell have been reported (albeit not yet confirmed) under cryogenic conditions or high pressure and were supported quantum-chemically (Wang et al., 2007; Botana et al., 2015; Miao et al., 2017; Gao et al., 2019; Pravica et al., 2019). Yet, mercury should not be included among the common transition elements, since all these higher-valent compounds are quite unstable in ambient conditions (Jensen, 2008).

The elements of groups 12 to 14 in period 7, namely copernicium, nihonium and flerovium (with lifetimes in the range of seconds) have electron configurations (6${\text{d}}_{5/2}^{4}$ 7s^2^ 7${\text{s}}_{1/2}^{0-2}$). As elementary substances, copernicium would be a volatile metallic noble liquid similar to mercury, and flerovium would be a volatile, rather noble metal (Yakushev et al., 2014; Schädel, 2015; Steenbergen et al., 2017; Mewes et al., 2019b). Little is known of their chemistry. Small molecules of low valency such as CnO, NhH, or FlF_2_ are similar to their lighter homologs, typically with slightly reduced bond strength (Liu et al., 2002; Demidov and Zaitsevskii, 2014, 2015).

An extreme chemical impact of scalar and spin-orbit relativistic effects occurs for the voltage of the mercury cell and in particular in lead batteries (Ahuja et al., 2011; Zaleski-Ejgierd and Pyykkö, 2011). Higher valences in period 7 were investigated by Ghosh and Conradie (2016). While HgF_4_ is at most meta-stable, the (*n*−1)d_5/2_ level of copernicium is sufficiently destabilized and the *n*s_1/2_ sufficiently stabilized, so that CnF_4_ becomes stable as D_4h_-Cn^4+^(6${\text{d}}_{5/2}^{4}$ 7s^0^) F_4_. In general, spin-orbit coupling weakens existing covalent bonding, because ligand field effects and spin-orbit effects perturb each other (Hafner et al., 1981), while here it induces bonding. A similar mechanism works six elements further, where the p-shell closure is delayed due to the destabilized (*n*-1)p_3/2_ level and the stabilized *n*s_1/2_ level, as described above. The d-shell closure happens in period 7 for flerovium, the 6d_5/2_ and 7s_1/2_ levels having moved down in energy (Figure 7). Eventually the 6d shell has become chemically inert, and the 7s-reluctant/inert-pair effect has grown. Consequently flerovium no longer has a raised valence as observed for roentgenium (VII) vs. gold (V), for copernicium (IV) vs. mercury (II) and possibly for nihonium (V) vs. thallium (III), but has instead the lowered valence of flerovium (II) vs. PbF_4_ (Ghosh and Conradie, 2016).

### The Late p-Block Elements

*The light elements O, F, Ne with core-like 2s*^2^ *shell are strongly electronegative with low valences 2, 1, 0; all heavier late p-elements are less electronegative but with higher valences 5 to 7 of the nsp shell. The superheavy members*_115_*Mc to*_118_*Og are more electropositive with rather stable 7s*½^2^*7p*½^2^ *and an active 7p*^3^*/*_2_ *valence shell without a large gap to 8s*½*. Little is known, but unusual chemistry is to be expected*.

The heavy elements of groups 15 to 18 have dominant valence electron configurations (*n*p_3/2_)^1−4^, while the *n*${\text{p}}_{1/2}^{2}$ and in particular the *n*${\text{s}}_{1/2}^{2}$ shells become more and more core-like. The first ionization potential, the electron affinity, and consequently the electronegativity too, of _115_Mc-7${\text{p}}_{3/2}^{1}$ and _116_Lv-7${\text{p}}_{3/2}^{2}$ are remarkably small (livermorium has also a small second ionization potential). The moscovium mono-halides have a strongly ionic character (Borschevsky et al., 2015; Santiago and Haiduke, 2020). The elements below the metal-non-metal divide in Figure 2-right are of a metallic character. The p-series in period 7 is mostly metallic, with astatine expected to be a metal (Hermann et al., 2013) and last member oganesson is either a semiconductor (Mewes et al., 2019a) or a metalloid, depending on one's definition (Vernon, 2013), or a metal (Gong et al., 2019). Trombach et al. (2019) has summarized the sparse chemical speculations, based on pronounced reluctant-pair and spin-orbit coupling effects.

## The Periodic Table as a Blinker as Well as an Eye-Opener

*There is a long history in chemistry about substances and reactions regarded as possible or impossible, according to powerful stories in journals and text-books. They emerged because periodicity expectations and other theoretical models excluded, precluded or prescribed them, and they were advanced by earlier accidentally unsuccessful experimental efforts causing accepted narratives in the community. This is another example of how heavily empirical observations and non-observations are theory-laden in positive and negative senses. Many compounds are metastable under ambient conditions, but only very specific synthetic routes yield them with low internal energy so that they will not decay over the activation barrier as soon as they are formed. These cases need to be distinguished from basically instable compounds that can only be kept near 0 K and if separated from other molecules (such as in cold noble gas matrices, or in the vacuum of mass spectrometers, or in molecular beams), or that are forced together by high pressures. One may construct a Periodic Table for the classification of (meta)stable compounds, or of unstable compounds at low T, or of unstable compounds hold together by high p. The properties of the elements of a group may appear more similar, if compared under more different and exotic conditions*.

### Blinkered Expectations Finally Verified

#### Group 18: Noble Gas Compounds

The unsuccessful attempts in the early twentieth century to prepare noble gas compounds were reviewed by Chernick (1963) and by Laszlo and Schrobilgen (1988). The inertness of the noble gases “was preached so dogmatically wherever chemistry was taught that few chemists would spend their time trying to produce *impossible compounds.”* The trends of valence at the ends of the 2nd vs. the later periods (namely 0 for Ne vs. 8 for Ar, Kr, Xe) are not consistent within the noble-gas group, and later turned out as unreliable (valences under ambient conditions are 0 for He and Ne; 1 and 2 for the border cases Ar and Kr; 6 or 8 for Xe; smaller for Rn; see Lozinšek et al., 2020; Rohdenburg et al., 2020). Partially correct experimental trials in the late 1920s and predictions by Pauling in the early 1930s were only reproducibly realized in the early 1960s. Extrapolations in the Periodic Table of both possibilities and impossibilities sometimes go wrong.

#### Group 17: Halogen Oxoacids

Similarly, while the perchlorates and periodates and their acids were long known, over a century passed between the first record of unsuccessful attempts to prepare *perbromic acid* (Watts, 1863) and its synthesis by Appelman in 1968 (see: Appelman, 1973). Greenwood and Earnshaw (Greenwood and Earnshaw, 1984 seq.; in particular 1998) later wrote that “The quest for perbromic acid and perbromates and the various reasons adduced for their apparent ‘non-existence’ make fascinating and salutary reading” including Pauling's and others' mispredictions (Herrell and Gayer, 1972). Another example is *hypofluorous acid*. In contrast to the customary heavier halogen oxyacids and their salts, no oxyacids of fluorine were known for a longtime. Thus, “chemists had pretty well-convinced themselves that no oxyacids of fluorine were ever likely to be isolated … [on the basis] of straightforward thermodynamic arguments.” Only in 1971, HOF was isolated as a chemical compound by Studier and Appelman (see: Appelman, 1973) and also LiOF as a molecule by Andrews and Raymond (1971).

#### Group 16: Chalcogen Chains

The heavier chalcogens from sulfur onward are known to form chains such as –S–S–S– etc. Concerning oxygen, only the monoxides _/_O_\_, peroxides _/_O–O^/^, and ozonides –O^/^O^\^O- (inorganic salts of O^−^, O^2−^, ${\text{O}}_{2}^{-}$, ${\text{O}}_{2}^{2-}$, ${\text{O}}_{3}^{-}$ and organic compounds) are well-known. However, the simple hydrogen trioxide, H_2_O_3_, already proposed by Berthelot (1880), was prepared in 1994 (Cerkovnik and Plesničar, 1993) and found to be metastable below −40°C. The tetroxide, H_2_O_4_, was suggested by Mendeleev in 1895 and characterized below −125°C (Levanov et al., 2011). Cryogenic conditions enable the realization of chemists' fantasies.

#### Group 15: Pentachlorides, and Chains

The existence of penta-halides of P and Sb in contrast to N, As, and Bi was one of the grounds for Biron (1915) to develop the concept of secondary vertical periodicity. Several explanations were subsequently put forward. Eventually Seppelt (1976, 1977) synthesized AsCl_5_ by irradiating a mixture of AsCl_3_ and liquid Cl_2_ with UV light at −105°C. However, AsCl_5_ decomposes at temperatures above −50°C. Single molecules NF_5_ and NCl_5_ are less stable even near 0 K, if at all (Bettinger et al., 1998). This is another case for the floating borderline between common environmental and exotic conditions at low T (or high p). *Polynitrogen:* Long-known small inorganic and organic poly-nitrogen compounds are the azides, containing the energetic anionic ${\text{N}}_{3}^{-}$ and radicalic ^∙^N_3_ species. The synthesis of higher nitrogen polymers as HEDMs (High Energy Density Materials) was unsuccessful for many decades, thus most chemists doubted during a century that such allotropic species of nitrogen could exist. Finally, the pentazonium chain cation ${\text{N}}_{5}^{+}$ was synthesized by Christe et al. (1999), and a cyclic aromatic ${\text{N}}_{5}^{-}$ pentazolate compound by Zhang et al. (2017). The elementary substances of the group 15 elements P to Bi are solid polymers under ambient conditions, while until recently nitrogen was only known as a dimeric gas. Predicted polymeric ‘black nitrogen’ phases are now established at high pressures and temperatures (Cheng et al., 2020; Ji et al., 2020; Laniel et al., 2020). The lightest member of group 15 is not that different, if very different conditions are compared.

#### Group 14: Oxoacids

Oxidation state IV is most common among group 4 and 14 elements, such as in the ubiquitous carbonates and silicates. Yet in 2012, Jespersen et al. (2012, p. 167, 180) wrote that “carbonic acid is too unstable to be isolated as a pure compound.” Pure carbonic acid had been isolated and characterized since 1990. It was even possible to sublime and re-condense the solid. While the activation barrier of the exothermic monomolecular decay, H_2_CO_3_ → CO_2_ + H_2_O, is as large as 2 eV, the process becomes auto-catalyzed by the water molecules (Hage et al., 1998; Loerting et al., 2000; Abramson et al., 2018).

#### Transition Groups 3–13

Lothar Meyer failed in the early 1860s (Meyer, 1864) with the chemical grouping of what we now call the transition elements. The vertical, horizontal, diagonal etc. similarity patterns are complex, and Mendeleev's success was bought by restraining the chemical view. The IUPAC (2012) and Connelly et al. (2005) defines the transition elements as the ones whose atoms may have a partially occupied d shell in their compounds, i.e., the nine groups 3–11. Group 3 elements have formally a d^1^ shell only in less common oxidation state II (Meyer, 2014), and group 11 element silver has an incomplete d^8^ shell only in less common oxidation state III. In contrast to the transition elements, the d-block elements are usually defined as comprising the 10 groups 3–12, where group 12 elements Zn, Cd, Hg have a closed d^10^ shell under ambient conditions. The inner transition elements are the 15 lanthanoids La to Lu and the 15 actinoids Ac to Lr, where the first ones (La, Ac) have an empty and chemically hardly active f^0^ shell, and the last ones (Lu, Lr) have a closed and chemically hardly active f^14^ shell.

#### Groups 1 and 2: Higher Valences

As mentioned in the subsection on the s-block elements, the heavier alkali metals can be multiply oxidized under high pressure. It has been theoretically predicted that Fr may behave as a typical mono-valent s-block element, but also as a polyvalent p-block element, breaking the Periodic Rule under ambient conditions. The art of synthesis that finally decides about the expected and unexpected gaps mentioned above, has here still to achieve the definitive answers.

### Periodically Unexpected Facts Finally Accommodated

*Only a fraction of the properties of the elements can be highlighted in any simplistic structure of Periodic Tables. Chemical handicrafts, scientific practice and theory only ‘incidentally’ discover the non-periodic physical and chemical properties of the elements, including the fuzzy end of the Periodic Table*.

#### Non-metal Diversity vs. Vertical Similarity Classification

**Figure 9:**
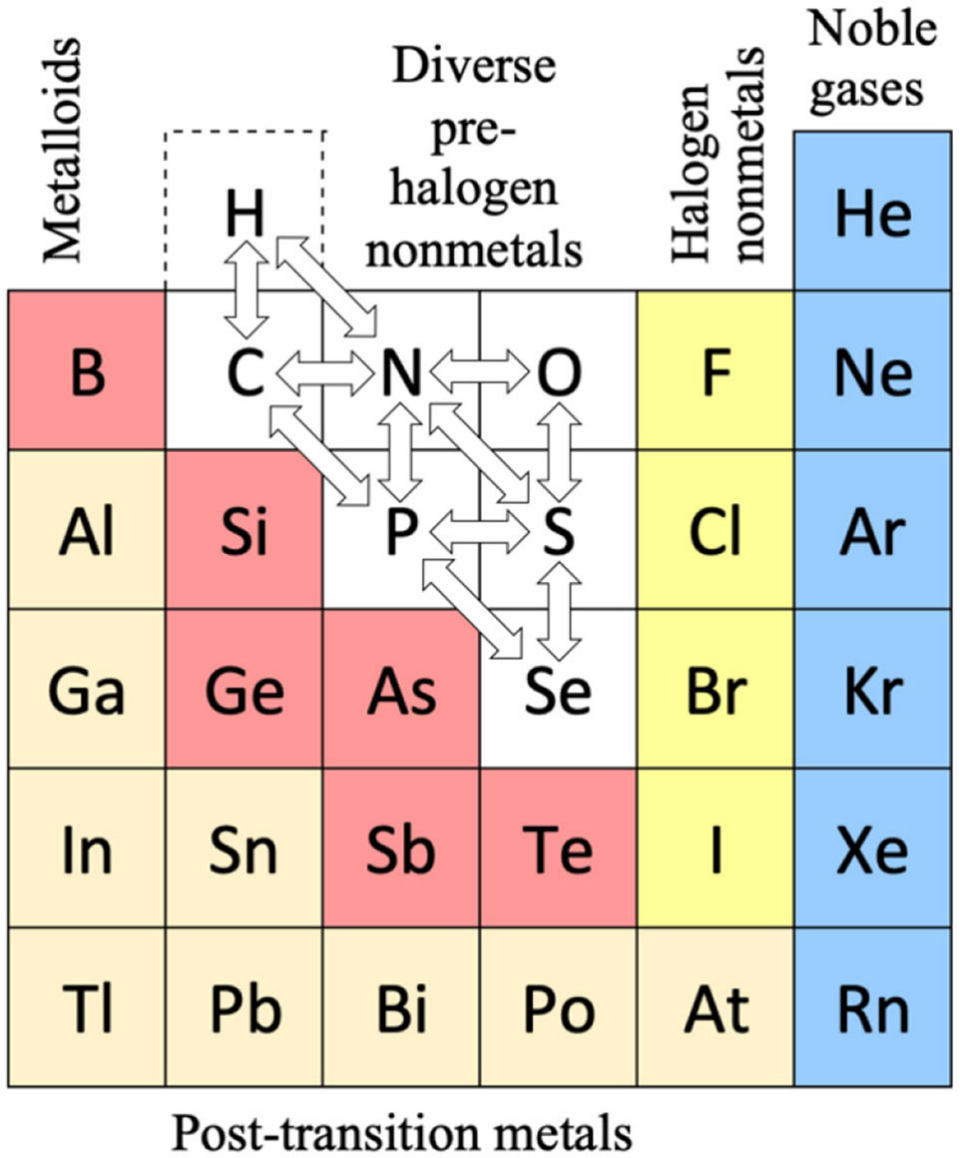
Periodic table extract, showing the non-metallic elements: the groups of noble-gases (blue) and halogens (yellow), and the pre-halogen non-metals including H (white), which are chemically quite diverse, with pronounced vertical, horizontal and diagonal relationships^1^ as recorded in the literature (Vernon, 2020). (No attempt is made here to quantify the strengths of the arrowed relations, nor are any cross-class relations considered). The post-transition metals (beige) and the “mysterious” metalloids (red) in between; Vernon (2013) are also displayed. Hydrogen (see footnote 2) in the dashed box, with 1s^1^ configuration, has one electron, one hole, and a half-filled shell, with intermediate electronegativity (EN); it is often placed above the alkali metals with one valence electron but low EN; sometimes above the halogens with one valence hole but high EN; rarely (here, compare Figure 2, Cronyn, 2003) above carbon with half-filled (2sp)^4^ shell and inter-mediate EN; and very rarely above boron which, like hydrogen, has one unpaired valence electron and very similar EN (Luchinskii and Trifonov, 1981). ^1^Many different stoichiometries and structures are known for each pair. For example, the nitrogen-oxygen pair with small electronegativity difference forms about two dozen of neutral and ionic molecular species N_1_${\text{O}}_{4}^{\text{q}}$ to N_4_${\text{O}}_{1}^{\text{q}}$. Among the less familiar ones are the peroxo-nitrate and ortho-nitrate anions N(O)_2_(O_2_)^1−^ and ${\text{NO}}_{4}^{3-}$, the trinitramide N(NO_2_)_3_, a possible rocket propellant (Lucien, 1958; Schulz et al., 1993; Goldstein and Czapski, 1998; Rahm et al., 2011; Anusha et al., 2018), and nitrosyl-azide ON-N_3_, a pale yellow solid stable below −50°C.

Aside from the noble gases and the halogens, the remaining nonmetals are often regarded as being too solitary and diverse to be discussed holistically in vertical groups (Figure 9). Metals can be gauged by their low values of electronegativity (or ionization energy and electron affinity; Yoder et al., 1975) *and* by the appearance of comparably diffuse orbitals in their atomic valence shells leading to broad metallic orbital bands, which result in the typical properties of metallic substances and the near-continuous variety of metallic elements. For the nonmetals Zuckerman and Nachod opined (Steudel, 1977) that “The marvelous variety and infinite subtlety of the non-metallic elements, their compounds, structures and reactions, is not sufficiently acknowledged in the current teaching of chemistry.” In fact the pre-halogen non-metals share more distinctive properties than any other class of elements. While the noble gases, as elemental substances, can be characterized by their invisibility and torpidity, and the halogens by their variegated appearance and acridity, the non-metallic pre-halogen elements exhibit the following characteristics: (i) being sandwiched between the strongly electronegative halogen nonmetals and the ‘weakly (non)metallic’ metalloids, their physical and chemical character is overall ‘moderately non-metallic’; (ii) the elemental substances have a semi-metallic [graphitic carbon, black phosphorus (the most stable form under ambient conditions, now easily prepared by Tiouitchi et al., 2019), selenium] or colored (sulfur) or colorless (hydrogen, nitrogen, and oxygen) appearance and possess a brittle comport-ment if in solid phase (including N under high pressure: Cheng et al., 2020; Ji et al., 2020; Laniel et al., 2020); (iii) they show an overall tendency to form covalent compounds featuring localized and catenated bonds as chains, rings, and layers; (iv) in light of their relatively small atomic radii and sufficiently low ionization energy values, a capacity to form interstitial and refractory compounds (West, 1931; Goldschmidt, 1967; Glasson and Jayaweera, 1968; Wulfsberg, 2000); (v) prominent geological, biochemical (beneficial and toxic), organocatalytic, and energetic aspects (Akerfeldt and Fagerlind, 1967; Hutzinger, 1980; Dalko and Moisan, 2004; Nancharaiah and Lens, 2015; Vernon, 2020a).

#### Metalloids as In-Between Elements

The different chemistry of all metals in s-, f-, d-, and p-blocks, and that of the “typical” non-metals from the upper-right p-block has largely been appreciated since the advent of modern chemistry. On ontological grounds, anything not metal-like is a non-metal, and this would include the metalloids found in the p-block (Oderberg, 2007). Since the metalloids (Halb-Metall in German) behave predominantly as chemically weak non-metals, the question arises: should we treat them simply in the class of otherwise nondescript nonmetals (Newth, 1894; Friend, 1914); or as a class *sui generis*. This is a typical example of the dependence of classification on the particular context, for instance whether Po, At or Rn shall be counted among the metalloids (Stein, 1985; Hermann et al., 2013). Concerning electric properties, the two metalloids germanium and silicon enabled the establishment of the semi-conductor industry in the 1950s and the development of solid-state electronics from the early 1960s (Vernon, 2013). Remarkable is the ‘diagonal’ range, overlaying the dominantly vertical structure of the chemical similarity groups, which however is not that unique in consideration of the diagonal relationship between the 2nd and 3rd periods (Edwards and Sienko, 1983; Greenwood and Earnshaw, 1984 seq.), the knight's move relationships (Rayner-Canham, 2020), and the ‘
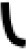
’ behavior of the closure of the p^6^ and d^10^ shells (Figure 1, right).

*Metallic Supercondictivity* at liquid-helium cooling temperatures and normal pressures was accidentally discovered in 1911 by Kamerlingh-Onnes for the metal that he could get in most pure form, Hg (Van Delft and Kes, 2010). In the following 75 years, many complex pure and doped substances were discovered that exhibit superconductivity within ca. 30 K; this appeared as an upper limit according to the Bardeen-Cooper-Schrieffer (BCS) theory. High-Temperature Supercondictivity (HTS) seemed impossible, and pushing the temperature higher was the stuff of fantasy. The discovery of HTS in 1986 came as an unexpected surprise. Since then many complex M^III^M^II^-cuprate materials such as (Y,La)Ba_2_Cu_3_O_7_ or TlBaCaCu_1.5_O_5_ have been discovered that become superconducting at liquid-nitrogen cooling temperatures (Kleiner and Buckel, 2016; Mangin and Rémi Kahn, 2017). Also materials becoming HTS under high pressure, including H_2_S, were found. Serendipity was of great relevance, while periodic trends of the two or three metallic partners in the copper oxides and the iron pnictides were just of partial help (Nipan et al., 2000; Kitazawa, 2012).

#### An End of the Periodic Table, Facts and Fantasy

Numerological arguments on the number and arrangement of elements in the periods led E. Q. Adams (1911) to the early supposition that elements of atomic weight greater than ca. 256 would not exist. In modern-day terms this equates to elements Z < 100 with lifetimes τ > 1 year, simple numbers Adams could not have dreamed of. Only astatine, radon, and francium are shorter-lived than _100_fermium with τ ≲ ¼ year. _99_Es and _100_Fm are the heaviest elements, which have been investigated in macroscopic quantities (Morss et al., 2010). For these heavy elements, bulk specimens such as crystals for x-ray structure analysis become quickly radiation damaged and may even evaporate. Accidentally, Adams' logically unfounded guess appears reasonable for today's practicing chemist. Remarkably, extrapolations of the non-relativistic structure of the periodicity of the upper part of the table into the region of ‘non-existing’ *Z* values of hundreds or thousands, violating published results on electronic structure of atoms up to the 170s (e.g., Pyykkö, 2011), are still published until these days.

## Summary and Conclusions

Physicists noted that the set of universal natural constants is fine-tuned within a narrow range. Thereby it allowed for the big-bang cosmic history, with the formation of a System of Elements of specific abundances, the formation of our sun and earth with a ‘habitable’ temperature-pressure range for some time period, allowing for life and the development of brains that can understand the big-bang cosmic history, with an *anthropocentric view of semantic consistency*.

Conservation principles are basic in physics. Modern scientific chemistry began with Lavoisier's law of the conservation of mass in chemical reactions. In chemistry the basic *conserved abstract entities are the elements*. The chemical element number *Z* is the physical *natural linear ordering parameter*, where *Z* determines the nuclear charge number and the atomic electron number in neutral chemical species; *Z* also determines all terms in the quantum-chemical Hamiltonian. As far as we know, the chemical elements behave strictly according to relativistic quantum theory. No indication of a theoretical defect is known at present concerning the simulation and explanation of chemistry under common conditions in this physical framework (Pyykkö, 2012a,b; Hettema, 2017; Schwerdtfeger et al., 2020). Of course there are many unsolved technical problems of solving the physical equations correctly. However, simplifying *ad-hoc* rules (such as the Periodic Rule, sometimes called the ‘Periodic law’) may show ‘exceptions from reality.’ Further, the electronic behavior of heavy-element systems at *the bottom of the present Periodic Table* causes deviations from the apparent periodicity that is showing up for the lighter elements. Accidentally also the nuclear lifetimes decrease at the bottom of the present Periodic Table so that there would anyway be no chemistry in the common sense in an extended table.

Chemistry is dominantly governed by the behavior of the valence-active electrons with orbital energies significantly above those of the atomic core shells. Every element differs from the other by *different core and/or valence shells*. Since the complicated effective screened potentials in atomic ions *Z*^*q*^^+^ deviate appreciably from the symmetric hydrogenic Coulomb potential, there appear *large orbital energy gaps above just filled 1s*^2^*, 2p*^6^ *to 5p*^6^ *or 6p*^6^*, and 3d*^10^ *to 5d*^10^ *shells*. The gaps vary along the *Z*-row and with effective atomic charge *q*+, therefore a fuzzy repetition of elemental qualities occurs at various steps of ΔZ. This causally complex phenomenon of chemical periodicity exhibits a somewhat *accidental structural symmetry*. Different aspects can be graphically highlighted. Various Periodic Tables for use in chemistry display aspects of our chemical knowledge, mostly referring to *common environmental conditions*.

Only a section of our chemical experiences can be approximately represented by a two-dimensional table, either flat or bent or split. The full chemical space is high dimensional. But the variations of several important chemical properties of the elements, in particular valence and maximum oxidation numbers (Riedel and Kaupp, 2009; Higelin and Riedel, 2017), effective atomic radii, electronegativity and metallicity, along the periods and down the groups are significantly correlated among each other (Kornilov, 1965), so that *a two-dimensional display is particularly knowledge-economic*.

Incorporating fashionable and exciting experiences under cryogenic or high-pressure conditions such as in outer space or inside the earth adds to the irregularities in the two-dimensional tabular projections, ‘deformed’ into a regular grid. The complex variation of chemical behavior of the elements at the top and bottom of the periodic system gives some clues and insight on chemistry under non-standard conditions, at very low temperatures or very high pressures. In the chemical sciences a pragmatic view of reality may result in Periodic Tables that are different from the dogmatic tables advocated in the meta-sciences.

The more or less approximate repetition of chemical properties along the array of elements ordered by their ordinal numbers *Z* is coupled to the closure and stabilization of the valence shells upon increasing *Z*. The s valence shell at the beginning of a period changes to the d(s) valence shell of the transition elements. The d valence shells in the two lowest periods are not drastically changed upon (f)^14^ shell filling (except for the lanthanoid and actinoid contractions, and d-f mixing for the early actinoids Pa to Pu). Upon (d)^10^ shell closure, the valence shell changes over to s(p) and then to (s)p type. The biggest change of orbital symmetry, energy, and radius of the valence shell occurs upon the *(sp)*^8^ *shell closure, which determines the periodicity*, its fix points and the property jumps from the halogens to the noble gases to the alkali metals.

Simplification is inherent in any periodic table. Yet the basic electronic outer-core and valence shells should be represented qualitatively correctly, concerning the relevant shell types, their electronic populations (see second line of Figure 6), *energies and radii* (see Figures 7, 8), which are both needed to understand the chemistry of the elements. If one wants to explain the structure of the Periodic System with the help of the orbital model, *two rules are inevitable for the orbital orders* in period *n*: for groups 0 and 1 (*n*−1)p^6^ < *n*s < *n*p < (*n*−1)d < (*n*−2)f; the nearly inverted rule for the majority of groups 4 or 5 to 18, (*n*−1)p^6^ < (*n*−2)f < (*n*−1)d < *n*s < *n*p; in between the d and f orbital collapses occur. *Free atoms in a physical vacuum and bonded atoms in chemical substances* are different objects. An atomic shell is chemically active under common conditions, if of intermediate energy and of intermediate radius. *Diffuse Rydberg orbitals* (see footnote 6) are important in atomic and molecular spectroscopy, they have some relevance for metallic band formation, but they are less important for covalent bonding. Conversely, *the 4f and later 5f orbitals are too small* in general, in the majority of cases, for covalent interaction [except for cerium, praseodymium, and thorium (?) to americium, in particular] but their energies are sufficient to support variable oxidation states.

First order rules or approximations can map the broad contours of the situation in chemistry. That said, the primogenic-kainosymmetric peculiarities at the top of the periodic system, the horizontal and vertical pseudo-periodicities over its body, and the modifications at the bottom due to both larger *n* and ℓ values and relativity, create a subtle and nuanced richness of chemistry that may not necessarily be encompassed by simple generalizations. Further experimental-chemical and theoretical-computational researches into the behavior of the full plethora of compounds of the elements remains required (Restrepo, 2018 has estimated the number of energetically stable chemical compounds as >10^60^, while chemists have so far explored only a negligible fraction of this huge chemical space), such as those that will follow in this issue.

## Author Contributions

All authors have written the manuscript and are responsible for the content.

## Conflict of Interest

The authors declare that the research was conducted in the absence of any commercial or financial relationships that could be construed as a potential conflict of interest.

## Footnotes

^1^We apply the word *atomic ‘shell’* for a single one-electron level such as *n*p^3^/_2_, and also for energetically adjacent sets of levels such as *n*p, or (*n*−1)d*n*s, or *n*sp. Note: orbitals with non-vanishing angular momentum such as p (ℓ = 1) or d (ℓ =2) are energetically split by relativistic spin-orbit coupling into two separate ℓ±½ spinor levels (see footnote 5). This phenomenon becomes qualitatively significant for the chemistry of the lower part of the periodic table. In particular, *n*p is split into two separate levels *n*p½ and *n*p^3^/_2_.

^2^The anomalous properties of hydrogen are due to its unique valence shell consisting of a single symmetric orbital, and to its unique core of a naked nucleus without inner screening electrons. The naked proton easily attaches itself to the nearest electron pair. Unlike the alkali metal (A) compounds, which can often be described as containing A^+^ units, hydrogen always tends to complete its 1s^2^ valence shell, with H^+^ rarely appearing in realistic descriptions. Hydrogen exhibits similarities with lithium in particular, because of the small 1s^2^ core of the 2nd period elements. Both hydrogen and lithium exhibit a significant *covalent* chemistry with slightly electronegative partners, while for compounds with strongly electronegative elements, the model of *ionic* interactions works well. Hydrogen atoms can stand in for alkali metal atoms in many alkali metal compounds. Hydrogen is also capable of forming alloy-like hydrides, featuring *metallic* bonding, with several transition metals.

^3^Sneath referred to two composite factors, which may be labeled *reactivity* (broadly related to oxidation potentials and electronegativity), and *metallicity*. Restrepo's two factors were (only) for binary compounds of the elements and their stoichiometries. Leach (also: Leach, 1999–2020) confirmed the importance of electronegativity as a basic elemental property, noting its strong correlation with numerous atomic parameters, physical, and chemical.

^4^For convenience, the yet less common *symbols of the super-heavy elements* are listed here. From *Z* = 113 to 118, the group numbers (in bold) of (f)d/sp elements happen to be just *g* = *Z*−100:

**Table:**

**Group**	**(3)/11**	**(3)/12**	**3/13**	**4/14**	**5/15**	**6/16**	**7/17**	**8/18**	**9/1**	**10/2**
(f)d block	_101_Md	_102_No	_103_Lr	_104_Rf	_105_Db	_106_Sg	_107_Bh	_108_Hs	_109_Mt	_110_Ds
sp block	_111_Rg	_112_Cn	_113_Nh	_114_Fl	_115_Mc	_116_Lv	_117_Ts	_118_Og	_119_Uue	_120_Ubn

^5^We apply the following *nomenclature conventions for orbitals*; “*Space-orbital*”: An *orbital* is a one-electron function in 3-dimensional space, ϕ(***r***), real or complex. Examples of p-shell ϕ are p_m = 0_ = p_z_ ~ z, p_m = +1_ = (p_x_+i·p_y_)/√2 ~ x+i·y. “~” means “proportional to.” “*Spin-orbital*”: Means ϕ(***r***)·α or ϕ(***r***)·β, where α and β indicate electrons with the same α or β spin everywhere in space. “α spin-up,” often symbolized by ↑, means an angular momentum vector with component +½ (in atomic units ħ) along the chosen reference axis, usually called *z*. The *x,y* component of the spin vector is √2 times larger. The angle of the spin vector with the reference axis is the ‘magic angle’ arcos√1/3 ≈ 54.7^o^ > 45^o^. An α spin-up points more to the side than up, ↗ or ↖! Since an angular momentum is of type ***r*** × ***p***, and the Heisenberg uncertainty principle holds for products of ***r***and ***p***, Δ***r***Δ***p*** ≥ **1**·ħ/2, orbital and spin angular momenta in 3-dimensional space have only 2 well-defined components. That is, in microscopic quantum-mechanical 3-dimensional reality, we cannot graphically describe an angular-momentum vector by a 3-component arrow, for sure not by a vertical arrow, but only, for instance, by a cone specified by the 2 parameters of height and opening angle. Then, a two-electronic spin-singlet state may be represented by anti-parallel arrows, e.g., ↗↙, while the three states of a spin-triplet may be symbolized by, for instance ↖↗, ↘↗, ↙↘. “*Spinor-orbital*”: In the real, relativistic world, the one-electron states are represented by spinors, consisting of two complex orbitals for the α and β contributions, ϕ_α_(***r***)·α+ϕ_β_(***r***)·β. For instance, the spin-orbit coupled ground-state of a p-electron may be described by spinor-orbital p_x_·β+i·p_y_·β+p_z_·α, or by its Kramer's mate p_x_·α−i·p_y_·α−p_z_·β. Both p½ spinor-orbitals and any mixture of them has a spherically symmetric density distribution.

^6^*Rydberg orbitals* are diffuse orbitals around an atom (or molecule), forming a series like the hydrogen-like orbitals with increasing quantum number *n*, their energies converging toward the ionization energy *IE* as *IE* – $Z_{\text{eff}}^{2}$/(*n*–δ_eff_)^2^ (in Rydberg energy units). They are too diffuse to contribute to local chemical bonds. This Rydberg must not be mixed up with the same name, more recently introduced by Weinhold and Landis (2005) in the framework of so-called natural bond orbital analysis for core and valence shell basis set remainder/garbage.

^7^*Orbital energy* ε is here defined as the negative atomic one-electron ionization energy. An atomic open shell configuration with ℓ > 0 may give rise to several to several hundred different energy levels, with a spread of several or many eV for neutral atoms (1 eV ≈ 10^5^ J/mol), and even more for cations (Wang et al., 2006; Schwarz, 2010b). Simplest example: the p^2^ valence configuration of group 14 elements has 15 states at five energy levels with a spread of ca. 200 (Si, Ge, Sn) to 260 (C) to 350 (Pb) kJ/mol. Note: It is an inappropriate chemical habit of specifying individual energies by only giving the originating electronic configuration without any *LSJp,i* state assignment. On the other hand, configurational average energies are more relevant anyway for chemical bonding than the somewhat “accidental” individual atomic ground state energies. Therefore we here define the orbital energies ε with respect to the configurational averages, which can be derived with the help of some theory, from spectroscopic observations or quantum computations.

^8^A ‘*leading configuration*’ is one with comparatively large weight: The quantum mechanical state *a* of an *n*-electron system is described by a wavefunction Ψ_*a*_(*x*_1_,…, *x*_n_), depending on all *i* = 1 to *n* electronic position-spin coordinates *x*_i_. Ψ_*a*_ can be expressed as an infinite sum of configurations of Pauli-antisymmetrized products of *n* spin(or)-orbitals (see footnote 5) each from ‘complete’ orbital set {ϕ_*r*_(*x*_i_), *r* = 1 to ∞}. There are basically three cases. (I) “The single-configuration model works”: one can find some set of *n* orbitals {ϕ_*q*_, *q* = 1 to *n*} so that just one orbital configuration well approximates the wave-function, Ψ_*a*_ ≈ A{Πϕ_*q*_(*x*_*i*_), *q*=1-*n, i*=1-*n*}; despite the same number of electrons, the two different concepts must not be mixed up. (II) “The state is strongly correlated”: several configurations *d* are needed for any acceptable approximation of Ψ_*a*_ ≈ Σ_*d*_ *c*_*d*_·A{Πϕ_*ad*_(*x*_*i*_)}; typical examples are the two-configurational Be atom 1s^2^2s^2^ and 1s^2^2p^2^ (Watson, 1960), or the transition metal complexes of high oxidation state such as the permanganate ion ${\text{MnO}}_{4}^{-}$ (Buijse and Baerends, 1990). Note: in both cases the set of orbitals can be mixed and transformed to another equivalent set, e.g., local or delocal orbitals (Autschbach, 2012). (III) “The orbital model breaks down”: the wavefunction Ψ cannot be reasonably approximated by any short sum Σ_*d*_. This happens frequently when an electron is ionized from the lower part of a valence or core band such as for ${\text{N}}_{2}^{+}$(2sσg^−1^) or Ca^+^(2s^−1^) (Cederbaum et al., 1977).

^9^*The (n*+ℓ*, n) rule applied* to the series of cations *Z*^+*q*^ of a given element *Z* (here chromium) up to the neutral atom *Z*^0^, is contrasted with the leading configurations of the ground states of the respective cations, either unbound in physical vacuum, or bound in chemical compounds:

**Table:**

	**Cr^**+6**^**	**Cr^**+5**^**	**Cr^**+4**^**	**Cr^**+3**^**	**Cr^**+2**^**	**Cr^**+1**^**	**Cr^**0**^**
Atomic ions according to the (*n*+ℓ, *n*) rule:	1s^2^-3p^6^	**4s**^**1**^	**4s**^**2**^	**3d**^**1**^**4s**^**2**^	**3d**^**2**^**4s**^**2**^	**3d**^**3**^**4s**^**2**^	**3d**^**4**^**4s**^**2**^
Real free atomic cations in physical vacuum:	1s^2^-3p^6^	**3d**^**1**^	**3d**^**2**^	**3d**^**3**^	**3d**^**4**^	**3d**^**5**^	**3d**^**5**^**4s**^**1**^
Real bound atoms in chemical compounds:	1s^2^-3p^6^	**3d**^**1**^	**3d**^**2**^	**3d**^**3**^	**3d**^**4**^	**3d**^**5**^	**3d**^**6**^* *The delocalized metallic valence bands give rise to some s orbital occupation of the transition metals, also an electronic surplus on formal metallic anions. However, the d- and f-block elements in the vast majority of metal complexes, and all the heavier p-block elements have the (*n*-1)d energetically below the *n*s orbital, violating the (*n*+ℓ, *n*) rule.

^10^*The crystal-field-MO or ligand-field model* well explains the chemistry and spectroscopy of transition metal compounds. The Lewis-basic pair-donating ligands form covalent-dative bonds with the d shell of the metal atom, which may back-donate d pairs if the ligands have low empty acceptor orbitals. A good approximation for the valence shell of a transition metal ion of formal charge *q* from element group *g* is by electron configuration d^*g*−*q*^. Note that the s shell plays hardly any role in real chemistry of d elements except for free or metallic or anionic d atoms. The highest occupied and lowest unoccupied orbitals of the complexes, responsible for redox and substitution reactions and optical effects, usually have significant d metal character. One distinguishes weak-field high-spin complexes with several near-degenerate singly-occupied d-type orbitals, and strong-field low-spin complexes (most common for the heavier d elements) with occupied lower d-type orbitals and unoccupied higher d-type orbitals above a gap.

^11^*The Two Cultures* of science and art were defined by physical chemist and novelist C.P. Snow in 1956/1959 (Snow, 1962). We interpret them as the fact and rationality; and the narrative and opinion based human endeavors (science & technology vs. humanities & social sciences). They become integrated in chemistry, for instance when periodic tables are designed on chemical properties for chemical education by the technique of the arts. Chemically different elements He and Be are positioned in Periodic Table 6 above to obtain a regular outer shape with elements therein classified according to the graphical similarly of the conventional symbols He-1s^2^ and Be-2s^2^, independent of the different quantum-mechanical meanings.
